# Cross-regulation by CrcZ RNA controls anoxic biofilm formation in *Pseudomonas aeruginosa*

**DOI:** 10.1038/srep39621

**Published:** 2016-12-21

**Authors:** Petra Pusic, Muralidhar Tata, Michael T. Wolfinger, Elisabeth Sonnleitner, Susanne Häussler, Udo Bläsi

**Affiliations:** 1 Department of Microbiology, Immunobiology and Genetics, Center of Molecular Biology, Max F. Perutz Laboratories, University of Vienna, Vienna Biocenter, Dr. Bohr-Gasse 9, 1030 Vienna, Austria; 2 Institute of Theoretical Chemistry, University of Vienna, Währingerstrasse 17, 1090 Vienna, Austria; 3 Center for Anatomy and Cell Biology, Medical University of Vienna, Währingerstrasse 13, 1090 Vienna, Austria; 4 Helmholtz Center for Infection Research, Molecular Bacteriology, Inhoffenstraße 7, 38124 Braunschweig, Germany

## Abstract

*Pseudomonas aeruginosa* (PA) can thrive in anaerobic biofilms in the lungs of cystic fibrosis (CF) patients. Here, we show that CrcZ is the most abundant PA14 RNA bound to the global regulator Hfq in anoxic biofilms grown in cystic fibrosis sputum medium. Hfq was crucial for anoxic biofilm formation. This observation complied with an RNAseq based transcriptome analysis and follow up studies that implicated Hfq in regulation of a central step preceding denitrification. CrcZ is known to act as a decoy that sequesters Hfq during relief of carbon catabolite repression, which in turn alleviates Hfq-mediated translational repression of catabolic genes. We therefore inferred that CrcZ indirectly impacts on biofilm formation by competing for Hfq. This hypothesis was supported by the findings that over-production of CrcZ mirrored the biofilm phenotype of the *hfq* deletion mutant, and that deletion of the *crcZ* gene augmented biofilm formation. To our knowledge, this is the first example where competition for Hfq by CrcZ cross-regulates an Hfq-dependent physiological process unrelated to carbon metabolism.

PA can form persistent biofilms in the lungs of CF patients^1^. Polymorphonuclear leukocytes are known to surround the biofilms in the CF lung and to consume the majority of O_2_ to produce reactive oxygen species, which suggested that PA biofilms may partially grow anaerobically in this environment^2^,^3^. Based on several studies, Yoon *et al*.^4^ provided first hints for anaerobic respiration in PAO1 biofilms. This has been recently supported through the quantification of compounds of the denitrification pathway in sputum of CF patients^5^.

In *Enterobacteriaceae*, the RNA chaperone Hfq is known to play a key role in riboregulation by facilitating the interaction between small regulatory RNAs (sRNAs) and their target mRNAs^6^. As a number of sRNAs have been shown to regulate virulence traits including biofilm formation in different bacterial pathogens^7^, the requirement for Hfq in RNA-mediated regulation most likely contributes to the observed attenuated virulence phenotype of respective *hfq* mutants^6^. In PAO1, a deletion of the *hfq* gene resulted in pleiotropic effects on growth and virulence^8^.

Although many putative regulatory RNAs have been identified in different PA strains^9^, the function of only a few has been revealed, and only three regulatory RNAs have been implicated in biofilm formation. The sRNA PhrS represents the founding member of anaerobically controlled PA sRNAs^10^. PhrS has been suggested to be involved in biofilm formation as it was shown to stimulate the synthesis of the Pseudomonas quinolone signal (PQS)^10^, which can induce the release of DNA that serves as a biofilm matrix component^11^. A stimulatory effect of PhrS on biofilm formation has been recently observed^12^. The PA protein binding RNAs RsmY and RsmZ antagonize the function of the translational regulator RsmA^13^. The RsmA protein is known to act as a translational repressor of *psl* mRNA, which prevents exopolysaccharide synthesis, and thus to control biofilm formation in a negative manner^14^. On the other hand, up-regulation of the RsmY/Z RNAs results in titration of RsmA, and therefore in increased biofilm formation^7^,^13^.

The aim of this study was to identify PA14 regulatory RNAs involved in anoxic biofilm formation, which is a poorly studied aspect of chronic PA infections. We show that the Hfq-binding RNA CrcZ is highly abundant under these conditions, and that it impacts on anoxic biofilm formation. Thus, in addition to its established role in carbon catabolite repression, where CrcZ acts as a decoy to abrogate Hfq-mediated translational repression of catabolic genes^15^, this study reveals a novel aspect of Hfq sequestration by CrcZ, that is cross-regulation of other Hfq-dependent physiological processes.

## Results and Discussion

**Figure 1:**
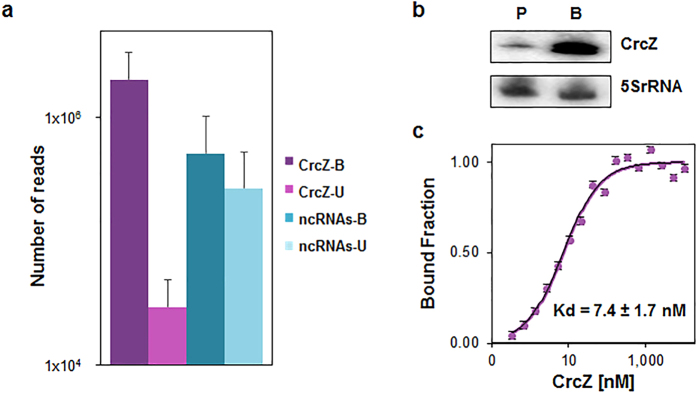
CrcZ is the major regulatory RNA bound to Hfq in anoxic biofilms. (**a**) Hfq-bound RNAs were isolated from lysates of B-96 cultures after co-immunoprecipitation with Hfq-specific antibodies. The identity of Hfq-bound and unbound RNAs in the supernatant was revealed by RNAseq. The number of reads for CrcZ in the Hfq-bound fraction (CrcZ-B; dark purple bar) and unbound fraction (CrcZ-U; light purple bar) is shown in comparison to the number of reads for all other described or putative PA14 regulatory RNAs in the Hfq-bound (ncRNAs-B; blue bar) and unbound fraction (ncRNAs-U; light blue bar). Error bars, mean ± s.d. of 2 biological replicates. (**b**) The steady state levels of CrcZ are elevated in B-96 cultures. The PA14 strain was grown in SCFM under aerobic conditions to an OD_600_ of 2 (P) and for 96 hours in anoxic biofilms (B), respectively. Total RNA was isolated and 10 μg of total RNA was used for the Northern-blot analysis. (**c**) K_d_ of Hfq for CrcZ RNA revealed by microscale thermophoresis. Increasing amounts of non-labelled *in vitro* transcribed CrcZ were added to 20 nM fluorescently labelled Hfq protein. The dissociation constant (Kd) of CrcZ was determined as described^47^, and was expressed as mean EC50 ± EC50 confidence interval of 2 independent experiments.

With the goal to identify regulatory RNAs that impact on anoxic biofilm formation, we concentrated on RNAs that interact with Hfq. The PA14 strain was grown in modified cystic fibrosis sputum medium (SCFM)^16^, which approximates to the conditions of the CF lung. Upon anaerobic biofilm growth of PA14 for 96 h in SCFM (B-96 cultures), Hfq-bound RNAs were isolated by co-immunoprecipitation with Hfq-specific antibodies. The identity of Hfq-bound and unbound putative and confirmed regulatory RNAs of PA14^17^ was revealed by RNAseq (Supplementary Table S1). All other reads were excluded from further analyses. Based on the total reads obtained for these RNAs (Hfq-bound and unbound), CrcZ was the most abundant PA14 regulatory RNA in B-96 cultures (Fig. 1a). The RNAseq results were mirrored by a Northern-blot analysis, showing that the levels of CrcZ RNA were ~50-fold higher in B-96 cultures than in planktonically grown PA14 cultures (OD_600_ = 2.0; P) (Fig. 1b). Moreover, the reads obtained for Hfq-bound CrcZ RNA outnumbered all other described or putative PA14 regulatory RNAs that interacted with Hfq by a factor of 4 (Fig. 1a). For verification, the Hfq-bound and unbound fractions were tested by RT-PCR for the presence of CrcZ RNA, ErsA RNA, which requires Hfq for function^18^, and RsmZ RNA, which poorly binds to Hfq^19^. Both, CrcZ and ErsA, were found in complex with Hfq, whereas the majority of RsmZ RNA was detected in the unbound fraction (Supplementary Fig. S1).

CrcZ transcription requires the alternative sigma factor RpoN and the response regulator CbrB, which is phosphorylated by the sensor/histidine kinase CbrA^20^,^21^. A recent transcriptome analysis indicated that the *cbrA* gene is 2.5-fold up-regulated in B-96 cultures when compared to P cultures^22^. This observation may partially rationalize the up-regulation of CrcZ in B-96 cultures. On the other hand, a putative motif for the anaerobic regulator Anr was identified upstream of the RpoN-dependent *crcZ* promoter (Supplementary Fig. S2). As *crcZ* was poorly expressed in a PA14*anr-* strain (Supplementary Fig. S2), it remains to be shown whether the observed up-regulation of *crcZ* in B-96 cultures (Fig. 1b) is indeed mediated by Anr.

As the intracellular quantities of a given Hfq-bound RNA depend on its abundance as well as on its affinity for the protein, we next determined the Kd of Hfq for CrcZ using microscale thermophoresis. The Kd was determined with ~7.4 nM (Fig. 1c). The high Kd of Hfq for CrcZ can be reconciled with six A-rich stretches in CrcZ^15^,^20^ to which Hfq can bind with its distal tripartite binding motifs^15^,^23^. The majority of Hfq was co-immunoprecipitated from the lysate of B-96 cultures (Supplementary Fig. S3) and CrcZ was the most abundant Hfq-binding RNA (Fig. 1a). Therefore, we next asked whether Hfq is crucial for and whether competition for Hfq by CrcZ can interfere with anoxic growth and biofilm formation.

To test whether Hfq is crucial for anaerobiosis, the growth of PA14 and PA14Δ*hfq* was monitored in SCFM. Under these conditions growth of the PA14Δ*hfq* was impaired (Supplementary Fig. S4). In addition, the metabolic activity of PA14 and PA14Δ*hfq* was assessed during anoxic growth in SCFM by employing a P*rrnB*P1*-gfp*(AGA) reporter gene^24^. When compared with strain PA14, the absence of Hfq resulted in a decrease of the GFP activity, indicating that PA14Δ*hfq* is less metabolically active than the wild-type strain (Supplementary Fig. S4).

These initial studies prompted us to perform a RNAseq based comparative transcriptome analysis with B-96 cultures of PA14 and the PA14Δ*hfq* mutant, respectively, to assess the impact of Hfq on anoxic biofilm formation in more detail. For the initial analysis a p-value (adjusted for multiple testing) of 0.05 was set as a threshold for statistical significance and the change in abundance (fold change) had to exceed ±4 for a given transcript in order to be considered differentially abundant. In addition, transcripts were also considered, if they were differentially abundant at least ±1.5-fold and part of an affected pathway or operon (Supplementary Table S2). 428 transcripts were found to be differentially expressed in the *hfq* mutant when compared with PA14; 296 and 132 transcripts showed at least a 4-fold increased and decreased abundance, respectively (Supplementary Table S2).

**Figure 2:**
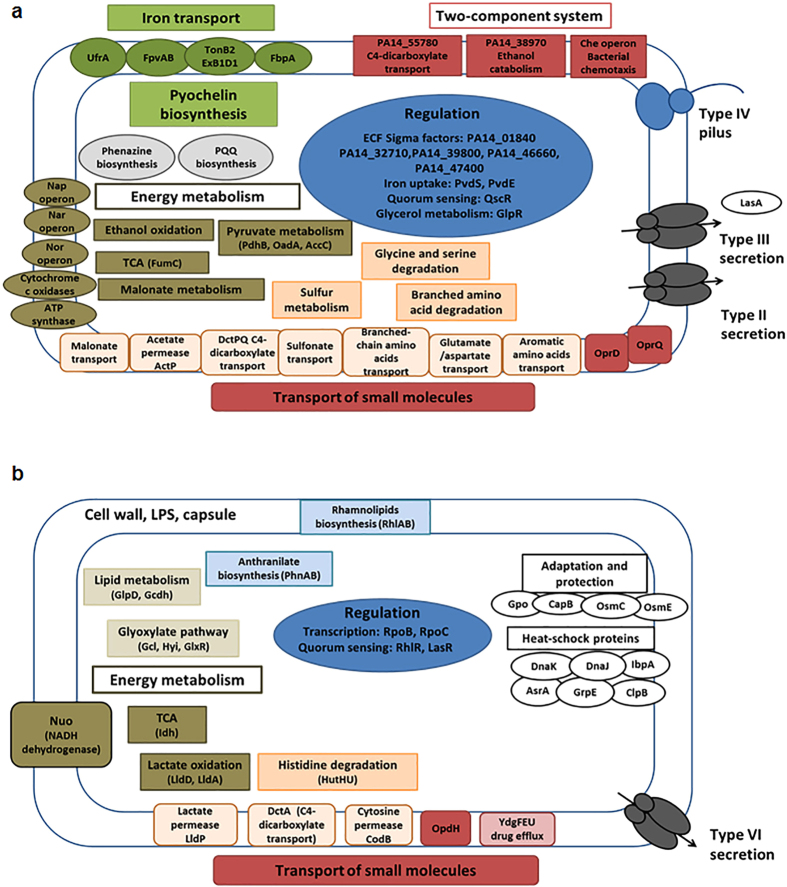
Hfq regulon in B-96 cultures. Regulatory and metabolic networks affected by Hfq. Increased (**a**) and decreased abundance (**b**) of the transcripts encoding for the corresponding proteins in PA14Δ*hfq* versus PA14. Functional classification according to the Pseudomonas Genome database^52^ and KEGG (Kyoto Encyclopedia of Genes and Genome)^53^.

These analyses revealed functions implicated in anoxic biofilm formation, which might be under direct or indirect control of Hfq (Fig. 2, Supplementary Table S2). Among them, the transcript encoding the quorum-sensing regulator QscR, known to act as a negative regulator of LasR and RhlR^25^, was 9.23-fold up-regulated in the absence of Hfq (Fig. 2a, Supplementary Table S2a). Accordingly, transcripts encoding the quorum-sensing regulators RhlR and LasR, as well as downstream genes involved in rhamnolipid biosynthesis (*rhlAB*) were down-regulated in the absence of Hfq (Fig. 2b, Supplementary Table S2b). As an impairment of QS can result in killing of PAO1 in anaerobic biofilms^4^, a de-regulation of the *qscR* gene in PA14Δ*hfq* could thus impact on anoxic biofilm formation.

The abundance of the transcript encoding glycerol-3-phosphate dehydrogenase GlpD, which serves as a major link between carbohydrate and lipid metabolism, was 4.84-fold decreased in the absence of Hfq (Fig. 2b, Supplementary Table S2b). This observation can be reconciled with the 11.51-fold increased abundance of the *glpR* transcript encoding the negative regulator of *glpD* (Fig. 2a, Supplementary Table S2a). As glycerol metabolism plays an important role in *P. aeruginosa* persistence by promoting biofilm formation^26^, a possible de-regulation of *glpD* in PA14Δ*hfq* might also interfere with anoxic biofilm formation.

PA can thrive under anaerobic conditions by acquiring ATP from glycolysis, pyruvate fermentation^27^, the arginine deiminase pathway^28^ as well as from denitrification^29^. When compared with PA14, the absence of Hfq resulted in increased abundance of transcripts encoded by the *nar, nap* and *nor* operons, encoding enzymes required for denitrification (Fig. 2a, Supplementary Table S2a). In contrast, the transcripts encoding the nitrite reductase (*nir*-operon) and the nitrous dioxide reductase (*nos* operon) did not show a differential abundance. However, a number of *nuo* transcripts, encoding subunits of the NADH dehydrogenase, were down-regulated in the absence of Hfq (Fig. 2b, Supplementary Table S2b). The NADH dehydrogenase is required for anaerobic growth in the presence of nitrate^30^. It contributes to the intracellular redox balance, i.e. the NADH/NAD^+^ ratio^31^, and its activity is coupled to the energizing processes of the membrane and ATP synthesis^32^, which in turn is required for sustained anoxic biofilm formation.

**Figure 3:**
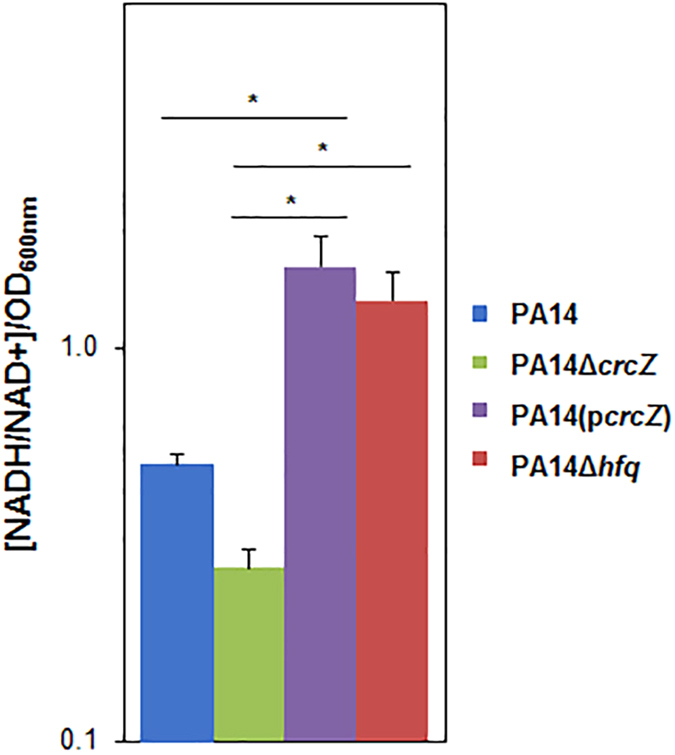
The absence of Hfq causes a redox imbalance. The NADH/NAD+ levels were assessed in B-96 cultures of PA14 (blue bar), PA14Δ*crcZ* (green bar), PA14(p*crcZ*) (violet bar) and PA14Δ*hfq* (red bar) grown in SCFM^48^. Error bars, mean ± s.d. N = 3 biological replicates with 3 technical replicates. *P < 0.05, analysed by one-way ANOVA with the Tukey’s HSD post hoc test.

A redox imbalance can impact on the fitness of bacteria^33^. As the transcriptome analysis indicated a lower abundance of some *nuo* transcripts as well as of other transcripts encoding dehydrogenases (*glpD*; *lldA*; *lldD;* Supplementary Table S2b) in the absence of Hfq, we next asked whether Hfq affects the intracellular NADH/NAD^+^ ratio. The NADH/NAD^+^ ratios were determined in B-96 cultures of strains PA14, PA14Δ*crcZ*, PA14(p*crcZ*) and PA14Δ*hfq*, respectively. When compared with strains PA14 and PA14Δ*crcZ*, an increase in NADH/NAD^+^ ratio was observed in PA14Δ*hfq* (Fig. 3). As the NADH/NAD^+^ ratio is crucial for the function of metabolic pathways, it seems reasonable to speculate that the reduced metabolic activity of the PA14Δ*hfq* strain (Supplementary Fig. S4) results at least in part from a redox imbalance.

Since the deletion of the *crcZ* gene augmented the metabolic activity of strain PA14Δ*crcZ* (Supplementary Fig. S4) and over-expression of a plasmid borne copy of *crcZ* mirrored both the anoxic growth phenotype and the increased NADH/NAD+ ratio of the PA14Δ*hfq* strain (Supplementary Fig. S4, Fig. 3), we next examined whether differential CrcZ levels affect anoxic biofilm formation. First, anoxic biofilm formation was assessed by a static crystal violet assay^34^ in B-96 cultures of the strains PA14, PA14Δ*crcZ*, PA14(p*crcZ*) and PA14Δ*hfq*, respectively. When compared with PA14, anoxic biofilm formation was increased in PA14Δ*crcZ*, whereas ectopic over-production of CrcZ (Supplementary Fig. S5) reduced anoxic biofilm formation to a level comparable with the PA14Δ*hfq* strain (Supplementary Fig. S5).

**Figure 4:**
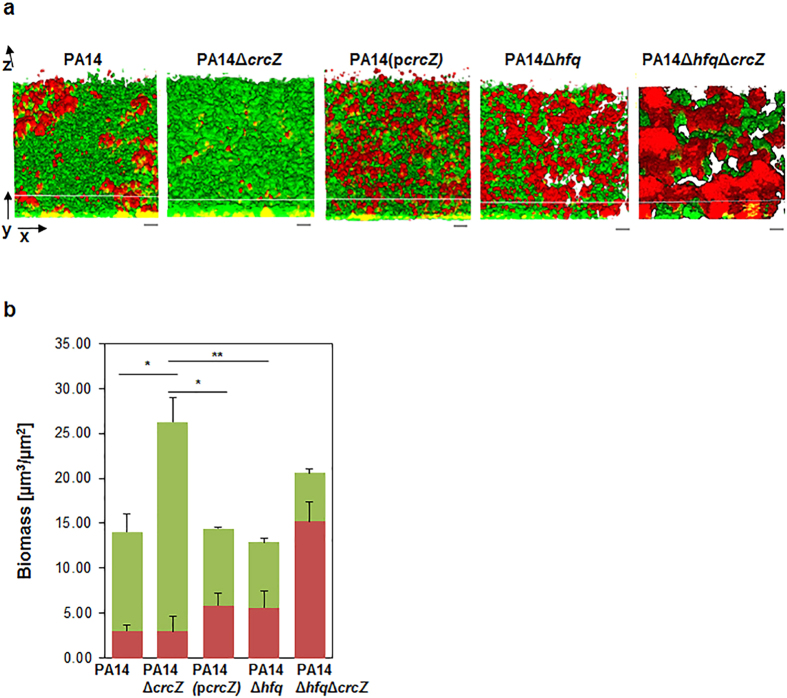
Biofilm formation of B-96 cultures of PA14, PA14Δ*crcZ*, PA14(p*crcZ*), PA14Δ*hfq* and PA14Δ*hfq*Δ*crcZ*. **(a)**The distribution of live and death cells in B-96 cultures of PA14, PA14Δ*crcZ*, PA14(p*crcZ*), PA14Δ*hfq* and PA14Δ*hfq*Δ*crcZ* were visualized by CLSM. Live cells (green) were stained with Syto 9 fluorescent dye, whereas dead cells (red) were visualized with propidium iodide. Three-dimensional images of biofilms (x, y, z-axes, scale bars represent 10 μm). The CrcZ levels in the different strains are shown in Supplementary Fig. S5a. (**b**) The biomass of live (green) and death (red) cells of B-96 cultures of PA14, PA14Δ*crcZ*, PA14(p*crcZ*), PA14Δ*hfq* and PA14Δ*hfq*Δ*crcZ* were quantified from CLSM vertical image stacks using Comstat2 software^51^. Error bars, mean ± s.d. from three independent experiments. *P < 0.05, **P < 0.01, analysed by one-way ANOVA with the Tukey’s HSD post hoc test.

Second, the viability and biomass of B-96 cultures of PA14, PA14Δ*crcZ*, PA14(p*crcZ*) and PA14Δ*hfq*Δ*crcZ* was assessed by live/death staining and visualized by confocal laser scanning microscopy (Fig. 4). When compared with PA14, a marked increase in both, the total and live cell biomass was observed for PA14Δ*crcZ* (Fig. 4). In contrast, when compared to PA14, an increase of dead cells was observed in anoxic biofilms of the CrcZ over-producing strain PA14(p*crcZ*) and of PA14Δ*hfq* (Fig. 4). Moreover, the PA14Δ*hfq*Δ*crcZ* displayed a marked increase in dead cell biomass and decrease of viable cells (Fig. 4). Taken together, these results corroborate the idea the competition for Hfq by CrcZ perturbs metabolic pathways, which results in diminished anoxic growth and biofilm formation in *P. aeruginosa*.

The CrcZ levels are known to increase in the presence of poor carbon sources or at a low carbon to nitrogen ratio^20^. Under these conditions, Hfq titration by CrcZ permits the uptake and utilization of non-preferred carbon sources^15^. When compared to planktonically grown cultures, the CrcZ levels were strongly increased in anoxic biofilms, which provides a further ambience where CrcZ can impact on Hfq function. Given the large Hfq regulon^19^ (Fig. 2) and the pivotal role of Hfq in PA physiology and virulence^8^, it appears worthwhile to explore *crcZ* regulation under a variety of conditions. A better understanding of the signals leading to CrcZ over-production could be valuable in view of developing strategies to attenuate the role of Hfq in PA pathogenicity.

CrcZ represents the first protein-binding RNA that limits biofilm formation. In contrast, several base-pairing sRNAs have been implicated in the regulation of biofilm formation in bacteria^7^. For example, the *Vibrio cholerae* VqmR RNA inhibits biofilm formation by translational silencing of the *vpsT* mRNA, encoding a transcriptional regulator of biofilm development^35^, whereas the *Salmonella* Typhimurium ArcZ RNA positively regulates *rpoS*^36^, which promotes biofilm formation^37^. Interestingly, over-expression of ArcZ RNA changed the profile of Hfq-bound sRNAs and mRNAs, which suggested that ArcZ titrates Hfq and thereby might also post-transcriptionally cross-regulate other genes^36^.

The biological function of CrcZ in limiting biofilm formation in PA might be associated with the complex environment in the CF lung. Recent studies indicated that polymorphonuclear leukocytes restrict the growth of PA in CF lungs, presumably by a high consumption of oxygen^38^. This could be linked with an increased CrcZ-mediated sequestration of Hfq under anoxic conditions and cessation of further biofilm formation, which in turn could be advantageous in terms of balancing the immune response and thus for establishing a long-term chronic infection.

## Methods

### Bacterial strains, plasmids and growth conditions

The strains and plasmids used in this study are listed in Supplementary Table S3. The synthetic cystic fibrosis sputum medium (SCFM) was prepared as described^16^. The concentration of FeSO_4_·7H_2_O was increased to 100 μM and that of KNO_3_ to 100 mM. This was done to allow for increased anaerobic biofilm formation after 96 h, which was required for the extraction of sufficient amounts of RNA for subsequent RNAseq analysis. For anoxic growth, 25 ml of bacterial cultures in SCFM were inoculated to an initial OD_600_ of 0.05 and then split in 1 ml aliquots into 5 ml polypropylene tubes. The cultures were incubated statically for 96 hours at 37 °C in a 2.5-liter anaerobic jar containing a gas pack (AN25; AnaeroGen, Oxoid, United Kingdom).

### RNAseq and CoIP RNAseq library construction and sequence analysis

The RNAseq analysis with total RNA prepared from two biological replicates of B-96 cultures was performed as recently described^22^. Hfq-bound RNAs were isolated from lysates of B-96 cultures after co-immunoprecipitation with Hfq-specific antibodies as described^15^. The Hfq-unbound fraction included all RNAs isolated from the supernatant after co-immunoprecipitation.

Total RNA from all samples was isolated using the TRIzol reagent (Ambion) according to the manufacturer’s instructions. The samples were DNase I treated, followed by phenol-chloroform-isoamyl alcohol extraction and ethanol precipitation. The Ribo-Zero rRNA Removal Kit (Gram-Negative Bacteria) Magnetic kit (Epicentre Biotechnologies) was used to deplete rRNA from samples used for RNAseq. Libraries were constructed using the NEBNext^®^ Ultra™ Directional RNA Library Prep Kit from Illumina. RNA sequencing has been performed at the Next Generation Sequencing Facility (VBCF, Vienna, Austria). The samples were subjected to Illumina 100 nt single end sequencing. PCR and index primers were removed from the raw reads with cutadapt^39^ and quality control was performed with FastQC^40^. Mapping of the samples against the PA UCBPP-PA14 reference genome (accession number NC_008463) was performed with segemehl^41^,^42^. Mapped sequencing data were split by strand and further processed for automatic UCSC Genome Browser^43^ visualization with the ViennaNGS toolbox^44^. Read counting for subsequent DESeq^45^ differential gene expression analysis was performed with BEDtools^46^. Only reads corresponding to putative or confirmed regulatory RNAs of PA14 were considered in the analysis.

### Northern-blot analyses

Total RNA was isolated by the hot phenol method as previously described^10^. The steady-state levels of CrcZ and 5 S rRNA (loading control) were determined using 10 μg of total RNA. The RNA samples were denatured for 10 min at 65 °C in loading buffer containing 50% formamide, separated on a 6% polyacrylamide/8 M urea gel, and then transferred to a nylon membrane by electroblotting. The RNAs were cross-linked to the membrane by exposure to UV light. The membranes were hybridized with gene-specific ^32^P-end-labelled oligonucleotides: K3 (CrcZ) and I26 (5 S rRNA), respectively (Supplementary Table S4). The hybridization signals were visualized using a PhosphorImager (Molecular Dynamics).

### *In vitro* transcription of CrcZ

For *in vitro* transcription of CrcZ the AmpliScribe T7-Flash Transcription Kit (Epicentre Biotechnologies) was used according to the manufacturer’s instructions. The 426 nt PCR fragment generated with the oligonucleotides E6 and C6 (Supplementary Table S4) served as template.

### Microscale thermophoresis

Specific binding between CrcZ and Hfq was determined by microscale thermophoresis (MST)^47^ using the Monolith NT.115 Green/Red apparatus (Nanotemper Technologies) at the Protein Technologies Facility (ProTech, VBCF, Vienna, Austria). Labelling of 20 μM of PA14 Hfq protein with NT-642 dye was performed using the Monolith™ Antibody Labelling Kit RED-NHS (Amine Reactive; Nanotemper Technologies) according to the manufacturer’s instructions. For the measurements, the concentration of the NT-642-labelled Hfq protein was kept constant (20 nM), whereas the concentrations of non-labelled *in vitro* transcribed CrcZ RNA varied from 1 to 1200 nM. The binding reactions were carried out in MST-buffer (10 mM HEPES pH 7.4, 150 mM NaCl, 10 mM MgCl_2_) supplemented with 0.1% Tween and 0.1% BSA. The reactants were initially incubated at 37 °C for 30 min to enable CrcZ binding with Hfq. The samples were then loaded onto NT.115 hydrophilic capillaries (Nanotemper Technologies). The data for microscale thermophoresis analysis were recorded at 25 °C using the red LED (excitation: 625 nm, emission: 680 nm); both, MST and LED Power at 40%. Data analyses were performed with NTAnalysis software.

### NADH assay

The [NADH]/[NAD^+^] ratios were determined in B-96 cultures using an enzyme cycling-based colorimetric assay^48^.

### Microscopy

Image acquisition was performed at the MFPL BioOptics Facility with a Zeiss LSM 700 confocal laser scanning microscope (Carl Zeiss, Germany) equipped with 405, 488, 555 and 639 nm laser diodes and two PMT channels for simultaneous monitoring of GFP (excitation, 488 nm; emission, 517 nm) and propidium iodide (excitation, 543 nm; emission, 565 nm). Images were obtained using a Plan-Apochromat 40x/1.3 Oil DIC objective. Simulated multichannel cross sections were generated using Fiji: an open-source platform for biological-image analysis and Volume Viewer Plugin^49^. The quantitative analyses of acquired image stacks of biofilms was performed using Comstat2 software^50^,^51^.

### Statistical analysis

All experiments were performed at least in duplicate. The data shown were obtained with three biological replicates, unless indicated otherwise. Statistical significance was determined by one-way ANOVA with the Tukey’s honestly significant difference (HSD) post hoc test when more than two groups with normal distribution were compared. Levene’s test was used to test for equality (homogeneity) of variances between tested groups. *P < 0.05, **P < 0.01 and ***P < 0.001 were considered statistically significant results.

### Data availability

The raw sequencing data were deposited in the NCBI sequence read archive (SRA) as a study under the accession number SRP062593 (RNAseq) and SAMN05823591 (CoIP RNAseq), respectively. All other data generated or analysed during this study are included within this article and the Supplementary information or are available from the corresponding author upon request.

## Additional Information

**How to cite this article**: Pusic, P. *et al*. Cross-regulation by CrcZ RNA controls anoxic biofilm formation in *Pseudomonas aeruginosa. Sci. Rep.* **6**, 39621; doi: 10.1038/srep39621 (2016).

**Publisher's note:** Springer Nature remains neutral with regard to jurisdictional claims in published maps and institutional affiliations.

## Supplementary Material

Supplementary Information
