# Supplementary material for: Cross-regulation by CrcZ RNA controls anoxic biofilm formation in Pseudomonas aeruginosa
Source: Sci Rep. 2016 Dec 21;6:39621. doi: 10.1038/srep39621 (PMC5175159; doi:10.1038/srep39621)
Supplement: Supplementary Information [file srep39621-s1.pdf]

Supplementary information

Cross-regulation by CrcZ RNA controls anoxic biofilm formation in *Pseudomonas aeruginosa*

Petra Pusic[1], Muralidhar Tata[1], Michael T. Wolfinger[2,3], Elisabeth Sonnleitner[1], Susanne Häussler[4] and Udo Bläsi[1*]

[1]Department of Microbiology, Immunobiology and Genetics, Center of Molecular Biology, Max F. Perutz Laboratories, University of Vienna, Vienna Biocenter, Dr. Bohr-Gasse 9, 1030 Vienna, Austria, [2]Institute of Theoretical Chemistry, University of Vienna, Währingerstrasse 17, 1090 Vienna, Austria, and [3]Center for Anatomy and Cell Biology, Medical University of Vienna, Währingerstrasse 13, 1090 Vienna, Austria, [4]Helmholtz Center for Infection Research, Molecular Bacteriology, Inhoffenstraße, 38124 Braunschweig, Germany

*Corresponding author.

Supplementary text

Construction of the PA14Δ*crcZ* mutant

An in-frame deletion of the *crcZ* gene was constructed by homologous recombination[54]. Briefly, the 730-bp upstream- and 717-bp downstream region of the *crcZ* gene was PCR-amplified using the primer pairs W100/Y100 and X100/Z100, respectively (Supplementary Table S4). The PCR products were linked by an overlapping PCR to generate a fragment containing the in-frame *crcZ* deletion. The fragment was cloned into the *Kpn*I and *Xba*I sites of plasmid pME3087 (Supplementary Table S3). Upon verification of the modification by DNA sequencing, the resulting plasmid was mobilized into PA14 with the aid of the *E. coli* strain HB101(pRK2013) and then chromosomally integrated through selection for tetracycline resistance. Excision of the vector by a second crossover event was achieved by enrichment for tetracycline-sensitive cells. The *crcZ* deletion was confirmed by standard molecular methods.

Construction of the PA14Δ*hfq*Δ*crcZ* mutant

A deletion of the *hfq* gene was introduced in PA14Δ*crcZ* mutant as described above. Briefly, the 717-bp upstream- and 799-bp downstream region of the *hfq* gene was PCR-amplified using the primer pairs H71/J71 and K71/I71, respectively (Supplementary Table S4). The PCR products were linked by an overlapping PCR to generate a fragment containing a 246-bp deletion, which spans the entire *hfq* coding region except for the stop codon. The fragment was cloned into the *BamHI* and *EcoRI* sites of plasmid pME3087 (Supplementary Table S3). Upon verification of the modification by DNA sequencing, the resulting plasmid was mobilized into PA14Δ*crcZ* with the aid of the *E. coli* strain HB101(pRK2013) and then chromosomally integrated through homologous recombination as described above. The *hfq* deletion was confirmed by standard molecular methods.

RT-PCR

RNA was extracted from B-96 cells after co-immunoprecipitation with Hfq-specific antibodies from the co-immunoprecipitate (CoIP, Hfq-bound fraction) and from the remaining supernatant (SN, Hfq-unbound fraction). For cDNA synthesis, 500 ng RNA template was mixed with 250 ng of random primers (Promega). The mixture was then treated at 65°C for 5 min, followed by 5 min incubation on ice. The cDNA synthesis was performed with AMV reverse transcriptase (Promega) according to the manufacturer´s instructions. 2 µl of cDNA derived from the RNA co-immunoprecipitated with Hfq and from the unbound total RNA, respectively, were used as a template for PCR amplification using the oligonucleotides C3 and E2 (Supplementary Table S4; CrcZ), T131 and U131 (Supplementary Table S4; ErsA) or D112 and E112 (Supplementary Table S4; RsmZ), respectively. The corresponding PCR products were 151 bp (CrcZ), 119 bp (ErsA) and 96 bp (RsmZ) in length. To confirm total DNA depletion of the RNA samples, the PCR reactions were performed with the same amount of RNA as used for the RT-PCR reaction without reverse transcriptase.

Western-blot analyses

Equal amounts of protein fractions were separated on 12.5% SDS-polyacrylamide gels and then electro-blotted to a nitrocellulose membrane. The blots were blocked with 5% dry milk in TBS buffer, followed by probing with rabbit anti-Hfq (Pineda). The antibody-antigen complexes were visualized with horseradish peroxidase conjugated secondary antibodies (Cell Signaling Technology) using the enhanced chemiluminescent (ECL) HRP substrate (SuperSignal West Pico Chemiluminescent Substrate, Thermo Scientific) and ChemiDoc Touch Imaging System (BioRad).

Metabolic activity assay using the rrnBP1-GFP$_{AGA}$ bioreporter

The delivery plasmid for miniTn7(Gm)-P$_{rrnBP1}$–*gfp*-AGA-a[24] and the helper plasmid pUX-BF13 (encodes the transposase genes) were introduced together by electroporation in strains PA14, PA14Δ*hfq* and PA14Δ*crcZ*, respectively, as previously described[55]. To monitor the metabolic state of the cells in anoxic biofilms, Gfp (excitation, 488 nm; emission, 517 nm) synthesis was measured in 24h-intervals for 4 days.

Biofilm assays

For the static crystal violet assay[34] the cultures were inoculated as described in the Methods section and incubated for 96h. Then, the contents of tubes were removed, washed 3 times with water and air dried. 1.2 ml of 0.1% (w/v) crystal violet was added followed by incubation at room temperature for 10 min. The tubes were washed and air dried. The stain attached to the tubes was solubilized using 95% ethanol. Biofilm formation was assessed by measuring the optical density of each sample at a wavelength of 595 nm.

Supplementary References

54. Ye, R. W. *et al.* Anaerobic activation of the entire denitrification pathway in *Pseudomonas aeruginosa* requires Anr, an analog of Fnr. *J. Bacteriol.* **177,** 3606–3609 (1995).

55. Koch, B., Jensen, L. E. & Nybroe, O. A panel of Tn7-based vectors for insertion of the *gfp* marker gene or for delivery of cloned DNA into Gram-negative bacteria at a neutral chromosomal site. *J. Microbiol. Methods* **45,** 187–195 (2001).

56. Gamper, M., Zimmermann, A. & Haas, D. Anaerobic regulation of transcription initiation in the *arcDABC* operon of *Pseudomonas aeruginosa*. *J. Bacteriol.* **173,** 4742–4750 (1991).

57. Liberati, N. T. *et al.* An ordered, nonredundant library of *Pseudomonas aeruginosa* strain PA14 transposon insertion mutants. *Proc. Natl. Acad. Sci. United States Am.* **103,** 2833–2838 (2006).

58. Wurtzel, O. *et al.* The single-nucleotide resolution transcriptome of *Pseudomonas aeruginosa* grown in body temperature. *PLoS Pathog.* **8,** e1002945 (2012).

59. Boyer, H. W. & Roulland-Dussoix, D. A complementation analysis of the restriction and modification of DNA in *Escherichia coli*. *J. Mol. Biol.* **41,** 459–472 (1969).

60. Voisard, C. *et al.* in *Molecular Ecology of Rhizosphere Microorganisms* 67–89 (Wiley-VCH Verlag GmbH, 1994). doi:10.1002/9783527615810.ch6

61. Figurski, D. H. & Helinski, D. R. Replication of an origin-containing derivative of plasmid RK2 dependent on a plasmid function provided in *trans*. *Proc. Natl. Acad. Sci. U. S. A.* **76,** 1648–1652 (1979).

62. Bao, Y., Lies, D. P., Fu, H. & Roberts, G. P. An improved Tn7-based system for the

single-copy insertion of cloned genes into chromosomes of Gram-negative bacteria. *Gene* **109,** 167–168 (1991).

Supplementary Figures

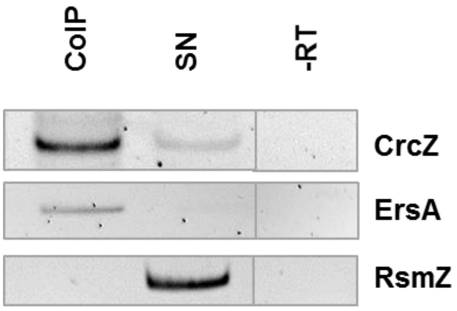

Supplementary Figure S1. Hfq co-immunoprecipitates with CrcZ and ErsA in B-96 cultures.

RNA was extracted from lysates of B-96 cultures after co-immunoprecipitation with Hfq-specific antibodies (CoIP; Hfq-bound fraction) and from the remaining supernatant (SN, Hfq-unbound fraction). Equal amounts were used for RT-PCR with specific primers for CrcZ, ErsA and RsmZ as described in Supplementary text. -RT, mock experiment with RNA obtained from the Hfq-bound fraction (CoIP) in the absence of reverse transcriptase.

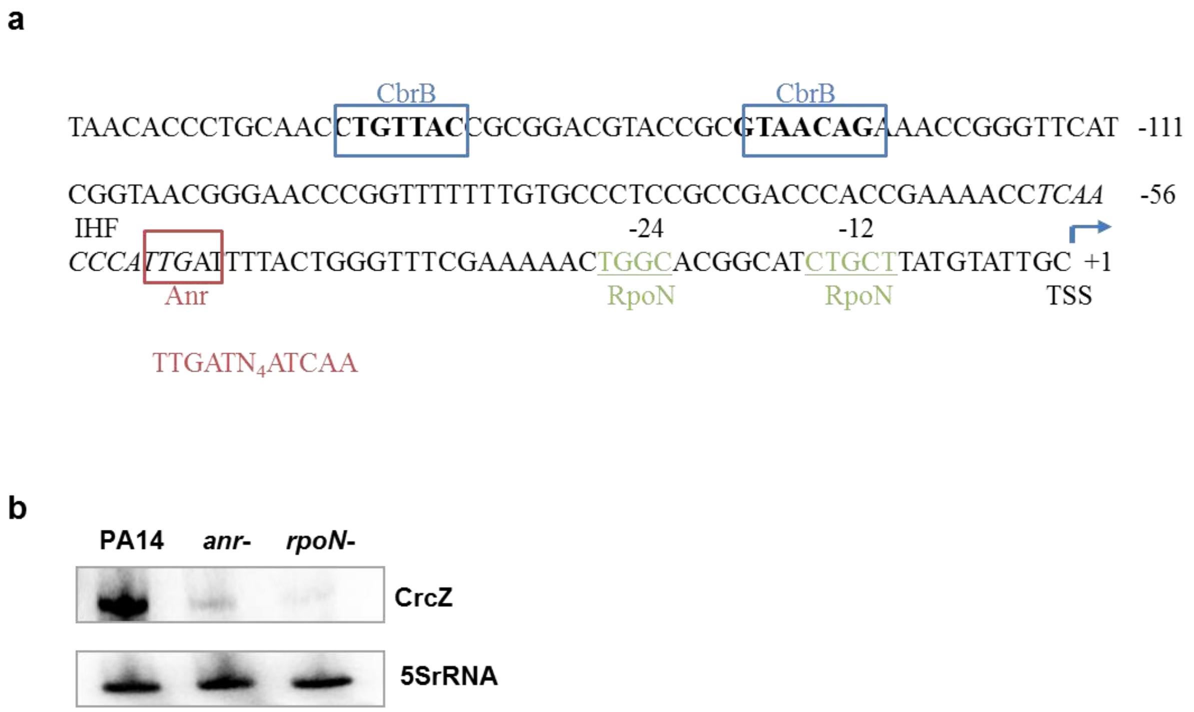

Supplementary Figure S2. Anr stimulates *crcZ* expression in B-96 cultures.

a Sequence of the *crcZ* promoter region. The transcriptional start site (TSS, +1) is marked with an arrow. The RpoN promoter sequence is shown in green and underlined. A putative IHF binding site is shown in italics. The CbrB binding sites and a partial putative recognition motif for Anr are shown by blue and red boxes, respectively[21]. The Anr consensus motif[56] is shown below.

b Total RNA was isolated from B-96 cultures of PA14, PA14*anr*- and PA14*rpoN*-, and 10 µg of total RNA was used for the Northern-blot analysis. 5S rRNA served as a loading control.

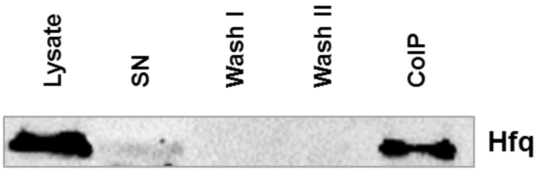

Supplementary Figure S3. Presence of Hfq in different steps during co-immunoprecipitation.

Samples were withdrawn from lysates of B-96 cultures during different steps of co-immunoprecipitation with Hfq-specific antibodies and examined by western-blotting for the presence of Hfq as described in Supplementary text. Lysate: initial lysate (input); SN (supernatant, Hfq-unbound fraction); wash I and II represent washing fractions; CoIP (co-immunoprecipitate, Hfq bound fraction).

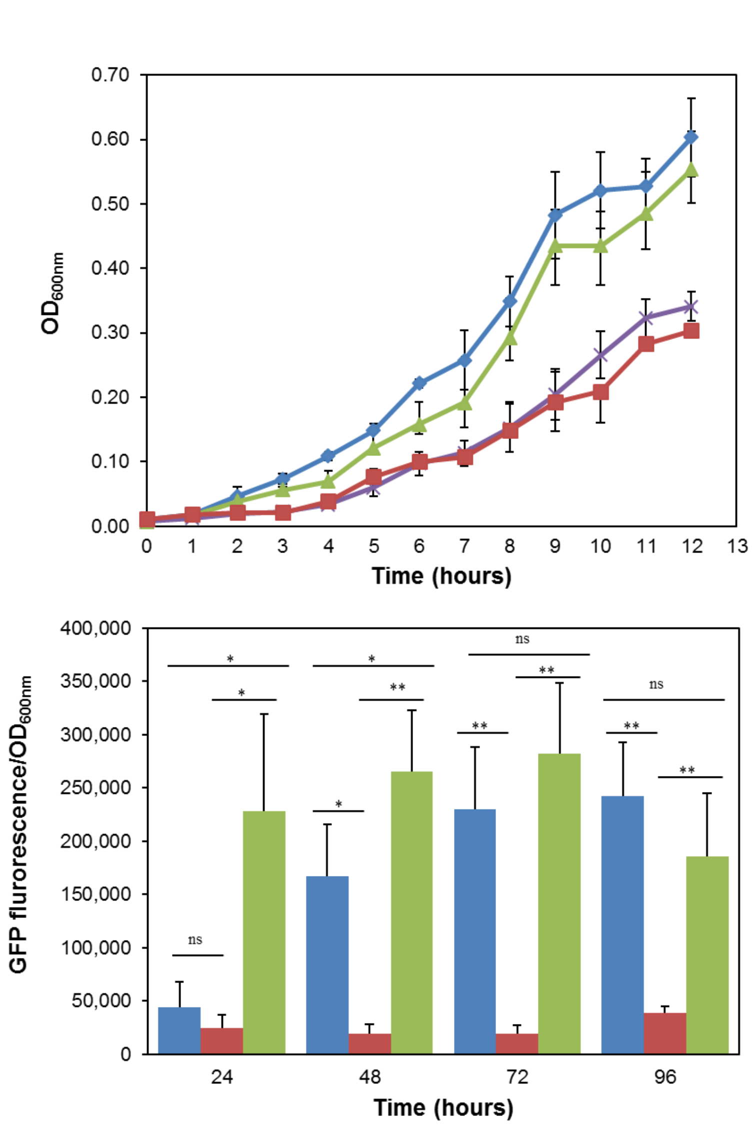

Supplementary Figure S4. Hfq is pivotal for anoxic growth and metabolism.

a The strains PA14 (blue bar), PA14Δ*crcZ* (green bar), PA14(p*crcZ*) (violet bar) and PA14Δ*hfq* (red bar), were grown anaerobically for 12 hours in SCFM. Cell growth was monitored by measuring the optical density at 600 nm (OD_{600nm}).

b Metabolic activity of strains PA14 (blue bar), PA14Δ*hfq* (red bar) and PA14Δ*crcZ* (green bar) monitored with the P*rrnB*P1-*gfp*(AGA) bioreporter after growth in SCF-medium. The synthesis of Gfp (excitation, 488 nm; emission, 517 nm), as well as the OD_{600} was measured every 24 h for 4 days. Error bars, mean ± s.d. from a representative of three independent

experiments. N = 3 biological replicates. ns (non-significant), * $P < 0.05$, ** $P < 0.01$, analysed by one-way ANOVA with the Tukey's HSD post hoc test.

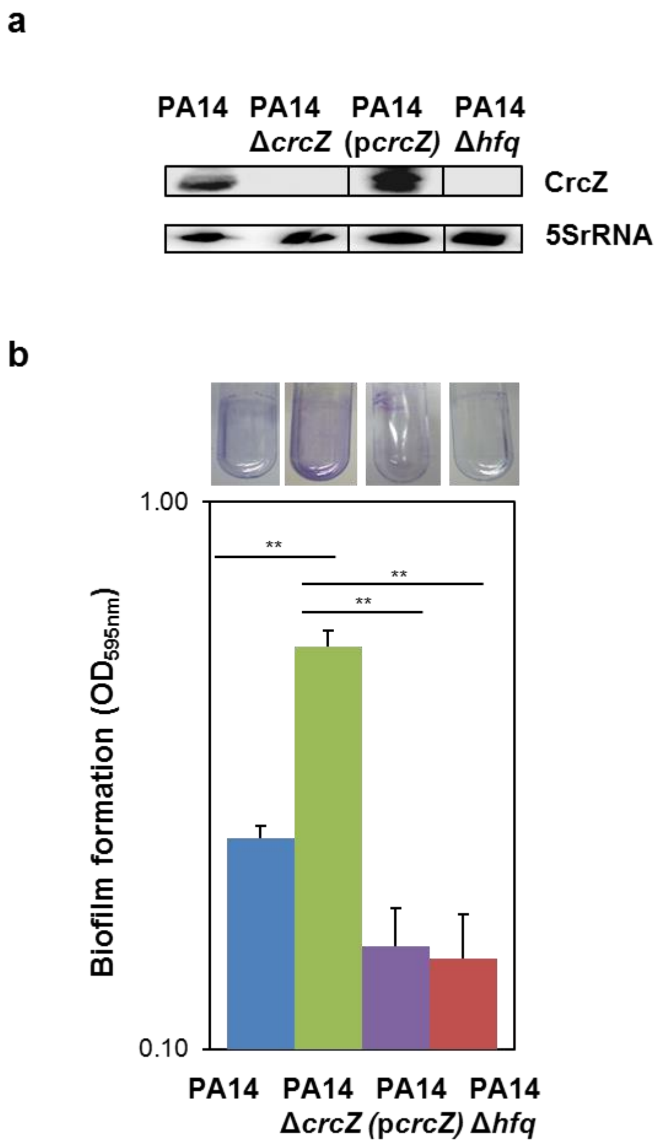

Supplementary Figure S5. Biofilm formation of B-96 cultures of PA14, PA14Δ*crcZ*, PA14 (p*crcZ*) and PA14Δ*hfq*.

a Steady-state levels of CrcZ in B-96 cultures of PA14, PA14Δ*crcZ*, PA14(p*crcZ*) and PA14Δ*hfq* (see also Fig. 4a). 10 µg of total RNA was used for the Northern-blot analysis with the CrcZ specific probe. 5SrRNA served as a loading control.

b Top, biofilm formation was assessed by a crystal violet assay. Bottom, graphical representation of the results obtained with B-96 cultures of PA14 (blue bar), PA14Δ*crcZ* (green bar), PA14(p*crcZ*) (violet bar) and PA14Δ*hfq* (red bar). Error bars, mean ± s.d. from a

representative of three independent experiments. N = 3 biological replicates with 3 technical replicates. ** $P < 0.01$, analysed by one-way ANOVA with the Tukey's HSD post hoc test.

Supplementary Table S1. Known and putative regulatory RNAs found in the Hfq-bound and unbound fraction. All RNAs with less than 150 reads were excluded.

sRNA_id[a]	Start[b]	End[c]	Strand[d]	Hfq bound[e] fraction	Hfq unbound[e] fraction
CrcZ_igr2625 (SPA0092)	5580518	5580924	+	2028048	29087
SPA0072 (tmRNA_ssrA)	4749699	4750100	+	368491	93138
SPA0094 (6SRNA_ssrS_igr2888)	6156499	6156800	+	45301	51387
SPA0091 (rnpB)	5119799	5120100	-	39037	25631
P30	5580674	5580896	-	24961	789
SPA0085 (RsmZ)	1500299	1500500	+	5413	70581
SPA0069 (RsmY)	596799	597000	+	4968	17203
SPA0011	2677699	2678200	+	2271	2425
SPA0012	3515399	3515700	+	2002	367
SPA0142	139699	140100	+	1582	64
SPA0104	1651699	1651900	+	1572	269
SPA0105	1648399	1649100	+	1475	51
SPA0021	5288099	5288500	+	848	257
rgsA_igr1076 (P16)	2250484	2250742	-	744	209
SPA0122 (ErsA)	6456399	6456600	-	734	89
SPA0076f (4.5S_igr1863)	3975599	3975800	-	637	1024
large_SRP	3975616	3975841	-	635	1024
SPA0124	4105199	4105600	-	623	657
PA14sr_035 (igr576)	1198079	1199053	-	608	171
PA14sr_090	3601259	3601463	-	560	42
igr1766	3758216	3764041	+	559	1
SPA0178	894999	895300	-	533	45
igr1944	4155629	4160668	+	522	1
SPA0145	1774199	1774400	-	518	24
SPA0013	3974899	3975200	-	461	20
SPA0025	2935699	2937400	-	358	2147
igr1276	2604153	2604747	-	346	51
SPA0147	2236399	2237000	-	279	63
SPA0126	1836399	1836900	-	250	3
igr329	642266	643346	-	231	13
SPA0073 (sRNA 645)	4444699	4445000	+	202	20
SPA0125	2464699	2465200	+	200	22
SPA0119	5951199	5952700	-	197	1
SPA0166	4085299	4085600	+	184	6
SPA0117	2154399	2154700	-	178	8
PA14sr_078	2924519	2925530	-	173	1
PA14sr_065	2465066	2465518	+	172	36
SPA0121	6444599	6445100	-	169	36

[a-d] Annotation based on location information according to[17]. [e] Sequencing reads averaged from two independent experiments.

Supplementary Table S2. Differential abundance of transcripts in B-96 cultures of PA14 and PA14Δ*hfq*.

a Transcripts with increased abundance in B-96 cultures of PA14Δ*hfq* when compared with PA14. A fold change cut-off of 4 and a p-value above 0.05 were applied. The colour coding of different functional classes corresponds to those shown in Fig. 2a.

PA14_id[b]	Gene[c]	Description[d]	Fold Change	log2Fold Change	pval	padj
**Adaptation, Protection**						
PA14_33690	*pvdE*	pyoverdine biosynthesis protein PvdE	4.05	2.02	1.83E-33	4.44E-32
PA14_49410		cold-shock protein	5.50	2.46	3.90E-58	2.37E-56
PA14_58000	*sodM*	superoxide dismutase	6.87	2.78	1.23E-20	1.54E-19
**Amino acid biosynthesis and metabolism**						
PA14_05750	*gltX*	oxidoreductase	17.34	4.12	9.97E-49	4.38E-47
PA14_37560	*asnB*	asparagine synthetase, glutamine-hydrolysing	7.84	2.97	1.77E-81	1.93E-79
PA14_69990	*dadX*	alanine racemase	7.04	2.82	8.54E-41	2.86E-39
PA14_71180		acetylornithine deacetylase	4.21	2.07	8.97E-12	6.13E-11
PA14_73090		hypothetical protein	6.56	2.71	1.71E-39	5.35E-38
**Glycine and serine degradation**						
PA14_32985	*gcvH2*	glycine cleavage system protein H	10.22	3.35	3.36E-62	2.25E-60
PA14_33000	*gcvP2*	glycine dehydrogenase	6.92	2.79	5.04E-75	4.72E-73
PA14_33010	*glyA2*	serine hydroxymethyltransferase	7.43	2.89	1.28E-72	1.11E-70
PA14_33030	*sdaA*	L-serine dehydratase	4.50	2.17	1.33E-41	4.55E-40
**Branched-chain amino acid degradation**						
PA14_18120	*mmsA*	methylmalonate-semialdehyde dehydrogenase	42.80	5.42	1.44E-227	1.69E-224
PA14_18140	*mmsB*	3-hydroxyisobutyrate dehydrogenase	74.19	6.21	5.91E-203	4.35E-200
PA14_35490	*lpdV*	dihydrolipoamide dehydrogenase	19.24	4.27	1.35E-142	3.32E-140
PA14_35500	*bkdB*	branched-chain alpha-keto acid dehydrogenase subunit E2	31.43	4.97	5.92E-152	1.66E-149
PA14_35520	*bkdA2*	2-oxoisovalerate dehydrogenase subunit beta	12.91	3.69	1.22E-49	5.47E-48
PA14_35530	*bkdA1*	2-oxoisovalerate dehydrogenase subunit alpha	7.36	2.88	9.55E-44	3.57E-42
PA14_19920		branched-chain alpha-keto acid dehydrogenase subunit E2	5.93	2.57	6.34E-35	1.63E-33
**Biosynthesis of cofactors, prosthetic groups and carriers**						
PA14_02420	*atsK*	hypothetical protein	55.33	5.79	9.83E-176	5.27E-173
PA14_13850	*moaA*	molybdenum cofactor	3.88	1.95	2.16E-09	1.17E-08

		biosynthesis protein A				
PA14_20140	*fpr*	ferredoxin--NADP+ reductase	7.80	2.96	3.12E-84	3.75E-82
PA14_31510		short-chain dehydrogenase	5.57	2.48	1.21E-20	1.53E-19
PA14_61750	*ipk*	4-diphosphocytidyl-2-C-methyl-D-erythritol kinase	5.51	2.46	2.08E-60	1.32E-58
PA14_66550	*hemE*	uroporphyrinogen decarboxylase	4.83	2.27	7.76E-38	2.25E-36
**PQQ biosynthesis**						
PA14_38780	*pqqE*	pyrroloquinoline quinone biosynthesis protein PqqE	6.55	2.71	2.45E-65	1.72E-63
PA14_38790	*pqqD*	pyrroloquinoline quinone biosynthesis protein PqqD	5.93	2.57	1.97E-46	8.06E-45
PA14_38800	*pqqC*	pyrroloquinoline quinone biosynthesis protein PqqC	5.73	2.52	1.07E-19	1.28E-18
PA14_38820	*pqqB*	pyrroloquinoline quinone biosynthesis protein PqqB	7.75	2.95	2.58E-71	2.14E-69
PA14_38825	*pqqA*	coenzyme PQQ synthesis protein PqqA	10.33	3.37	1.15E-13	9.14E-13
		**Carbon compound catabolism**				
PA14_02760	*catI*	CoA transferase, subunit A	4.04	2.01	9.05E-10	5.13E-09
PA14_20560	*amiE*	acylamide amidohydrolase	6.29	2.65	7.06E-56	3.96E-54
PA14_32160	*antA*	anthranilate dioxygenase large subunit	8.73	3.13	1.83E-25	2.97E-24
PA14_37550		ring-hydroxylating dioxygenase small subunit	8.30	3.05	7.68E-44	2.90E-42
PA14_37570		ring-hydroxylating dioxygenase, large terminal subunit	14.33	3.84	1.79E-127	3.90E-125
PA14_38590	*bdhA*	3-hydroxybutyrate dehydrogenase	6.38	2.67	1.60E-64	1.11E-62
PA14_70640	*rubA1*	rubredoxin 1	6.91	2.79	3.50E-53	1.82E-51
**Malonate metabolism**						
PA14_02580	*mdcD*	malonate decarboxylase subunit beta	4.55	2.19	9.33E-17	9.36E-16
PA14_02590	*mdcE*	malonate decarboxylase subunit gamma	8.21	3.04	2.05E-52	1.01E-50
		**Central intermediary metabolism**				
PA14_09290	*pchG*	pyochelin biosynthetic protein PchG	3.89	1.96	4.08E-11	2.61E-10
PA14_36330	*hcnA*	hydrogen cyanide synthase HcnA	4.30	2.10	9.90E-33	2.31E-31
PA14_40040		hypothetical protein	60.18	5.91	8.52E-197	5.57E-194
PA14_46960	*ggt*	gamma-glutamyltranspeptidase	4.13	2.05	7.56E-24	1.13E-22
**Sulfur metabolism**						
PA14_02310	*atsA*	arylsulfatase	23.06	4.53	1.76E-154	5.45E-152
PA14_40770	*cysI*	sulfite reductase	4.43	2.15	2.60E-46	1.06E-44
		**Chaperones & heat shock proteins**				
PA14_20570	*amiB*	chaperone	7.39	2.88	8.11E-36	2.18E-34

		**Chemotaxis**				
PA14_55960	*pctC*	chemotactic transducer PctC	5.93	2.57	3.73E-08	1.78E-07
**Che operon**						
PA14_02180	*cheB*	chemotaxis-specific methylesterase	8.09	3.02	6.74E-32	1.52E-30
PA14_02190	*cheD*	hypothetical protein	16.43	4.04	3.07E-57	1.85E-55
PA14_02200	*cheR*	chemotaxis protein methyltransferase	16.67	4.06	5.26E-83	6.08E-81
PA14_02220		chemotaxis transducer	13.31	3.73	3.76E-114	6.71E-112
PA14_02230	*cheW*	purine-binding chemotaxis protein	12.38	3.63	9.85E-73	8.66E-71
		**DNA replication, recombination, modification and repair**				
PA14_70600	*hupA*	HU family DNA-binding protein	3.92	1.97	1.51E-07	6.84E-07
		**Energy metabolism**				
PA14_65080	*ygiR*	hypothetical protein	3.95	1.98	2.26E-37	6.37E-36
PA14_65810		hypothetical protein	6.22	2.64	5.39E-23	7.61E-22
PA14_02450		NAD(P) transhydrogenase subunit alpha part 1	4.69	2.23	8.67E-30	1.80E-28
PA14_02470	*pntB*	pyridine nucleotide transhydrogenase subunit beta	4.95	2.31	2.78E-29	5.57E-28
PA14_02680	*aldH*	aldehyde dehydrogenase	15.71	3.97	4.76E-17	4.86E-16
PA14_38750		iron-containing alcohol dehydrogenase	5.68	2.51	7.86E-60	4.87E-58
**Pyruvate metabolism**						
PA14_19910	*pdhB*	pyruvate dehydrogenase E1 component, beta chain	4.23	2.08	6.05E-13	4.54E-12
PA14_71720	*oadA*	pyruvate carboxylase subunit B	6.54	2.71	8.97E-67	6.77E-65
PA14_71740	*accC*	acetyl-CoA carboxylase biotin carboxylase subunit	8.12	3.02	2.75E-53	1.45E-51
**Cytochrome c oxidases**						
PA14_01300	*coxA*	cytochrome c oxidase subunit I	6.51	2.70	1.22E-46	5.11E-45
PA14_01310	*coxG*	cytochrome C oxidase assembly protein	10.69	3.42	5.89E-38	1.72E-36
PA14_01320	*coIII*	cytochrome c oxidase subunit III	8.67	3.12	8.97E-54	4.81E-52
PA14_40510	*ccoN-2*	cbb3-type cytochrome c oxidase subunit I	9.35	3.23	2.62E-37	7.36E-36
PA14_44340	*ccoN*	cbb3-type cytochrome c oxidase subunit I	8.50	3.09	1.47E-82	1.67E-80
PA14_44350	*ccoO*	cbb3-type cytochrome c oxidase subunit II	13.84	3.79	2.69E-102	4.06E-100
PA14_44360	*ccoP*	cytochrome c oxidase, cbb3-type subunit III	10.57	3.40	4.34E-89	5.56E-87
PA14_44370	*ccoN*	cbb3-type cytochrome c oxidase subunit I	5.27	2.40	2.07E-37	5.89E-36
PA14_45330	*ccmE*	cytochrome c-type biogenesis protein CcmE	3.97	1.99	1.71E-05	6.02E-05
PA14_57560		cytochrome b	4.06	2.02	7.85E-41	2.64E-39
PA14_60700	*ccpR*	cytochrome c551 peroxidase	6.39	2.68	2.51E-54	1.37E-52

173

**Ethanol oxidation**						
PA14_38840	*exaC*	NAD+ dependent acetaldehyde dehydrogenase	127.05	6.99	7.40E-255	1.45E-251
PA14_38850	*exaB*	cytochrome c550	169.04	7.40	6.78E-160	2.35E-157
PA14_38860	*exaA*	quinoprotein alcohol dehydrogenase	260.81	8.03	0.00E+00	0.00E+00
**Denitrification (*nor* operon; nitric-oxide reductase)**						
PA14_06810	*norC*	nitric-oxide reductase subunit C	3.89	1.96	5.89E-19	6.76E-18
PA14_06830	*norB*	nitric-oxide reductase subunit B	4.47	2.16	8.15E-38	2.35E-36
PA14_06840	*norD*	dinitrification protein NorD	4.64	2.21	9.57E-11	5.95E-10
PA14_11690	*ppa*	inorganic pyrophosphatase	3.98	1.99	2.21E-13	1.72E-12
**Denitrification (*nar* operon; membrane-bound nitrate reductase)**						
PA14_13780	*narG*	respiratory nitrate reductase alpha subun	4.53	2.18	4.04E-45	1.61E-43
PA14_13800	*narH*	respiratory nitrate reductase beta subuni	5.35	2.42	4.42E-53	2.28E-51
PA14_13810	*narJ*	respiratory nitrate reductase delta chain	4.47	2.16	1.32E-30	2.85E-29
PA14_13830	*narI*	respiratory nitrate reductase gamma chain	5.20	2.38	2.43E-38	7.25E-37
**Denitrification (*nap* operon; periplasmic nitrate reductase)**						
PA14_49210	*napE*	periplasmic nitrate reductase NapE	4.34	2.12	1.13E-27	2.03E-26
PA14_49230	*napD*	NapD protein of periplasmic nitrate reductase	4.03	2.01	1.46E-09	8.07E-09
PA14_49250	*napA*	nitrate reductase catalytic subunit	7.81	2.96	2.87E-69	2.32E-67
PA14_49260	*napB*	cytochrome c-type protein NapB precursor	11.05	3.47	1.72E-40	5.66E-39
PA14_49270	*napc*	cytochrome c-type protein NapC	11.71	3.55	1.41E-56	8.31E-55
**TCA cycle**						
PA14_58030	*fumC*	fumarate hydratase	4.17	2.06	1.12E-42	3.95E-41
**ATP-synthase**						
PA14_73310	*atpB*	F0F1 ATP synthase subunit A	4.03	2.01	4.45E-40	1.43E-38
PA14_73320	*atpI*	F0F1 ATP synthase subunit I	4.85	2.28	2.88E-48	1.23E-46
**Fatty acid and phospholipid metabolism**						
PA14_18150	*acsL*	acetyl-coa synthetase	15.93	3.99	9.47E-136	2.23E-133
PA14_31530		acyl-CoA thiolase	4.96	2.31	6.03E-07	2.55E-06
PA14_39990		hypothetical protein	113.00	6.82	1.01E-252	1.49E-249
PA14_65820		acyl-CoA dehydrogenase	7.40	2.89	1.07E-11	7.24E-11
PA14_69660	*lppL*	lipopeptide LppL	4.34	2.12	4.33E-05	1.46E-04
**Hypothetical, unclassified, unknown**						
PA14_01340		hypothetical protein	9.41	3.23	1.41E-35	3.71E-34
PA14_04020		hypothetical protein	5.99	2.58	1.99E-40	6.48E-39
PA14_05110		hypothetical protein	5.30	2.41	1.56E-12	1.14E-11
PA14_07355		hypothetical protein	37.61	5.23	1.88E-186	1.11E-183
PA14_07370		hypothetical protein	5.44	2.44	1.31E-29	2.64E-28

PA14_07650	*ycgB*	SpoVR family protein	6.12	2.61	2.13E-32	4.88E-31
PA14_07660		hypothetical protein	4.55	2.18	6.62E-43	2.38E-41
PA14_13920		hypothetical protein	4.14	2.05	5.32E-15	4.68E-14
PA14_14540		hypothetical protein	3.98	1.99	2.28E-23	3.33E-22
PA14_20840		hypothetical protein	4.00	2.00	5.10E-25	8.09E-24
PA14_28390		hypothetical protein	10.41	3.38	9.75E-42	3.38E-40
PA14_29470		hypothetical protein	6.22	2.64	9.62E-43	3.41E-41
PA14_34050		hypothetical protein	4.89	2.29	1.49E-18	1.66E-17
PA14_34070		hypothetical protein	3.90	1.96	9.37E-24	1.39E-22
PA14_34080		hypothetical protein	4.08	2.03	7.09E-10	4.06E-09
PA14_34100		hypothetical protein	4.19	2.07	1.42E-09	7.86E-09
PA14_34130		hypothetical protein	5.29	2.40	1.36E-46	5.65E-45
PA14_37410		hypothetical protein	3.87	1.95	2.35E-25	3.80E-24
PA14_37520		hypothetical protein	4.83	2.27	2.27E-28	4.27E-27
PA14_38880		hypothetical protein	505.05	8.98	6.91E-175	3.13E-172
PA14_38920		hypothetical protein	29.89	4.90	5.09E-124	1.07E-121
PA14_38990		hypothetical protein	32.29	5.01	1.70E-170	6.69E-168
PA14_39000		hypothetical protein	58.99	5.88	6.05E-20	7.36E-19
PA14_40010		hypothetical protein	155.00	7.28	8.30E-271	2.44E-267
PA14_40020		hypothetical protein	46.22	5.53	2.12E-175	1.04E-172
PA14_40050		hypothetical protein	38.89	5.28	8.11E-112	1.40E-109
PA14_40100		hypothetical protein	4.94	2.30	6.08E-30	1.28E-28
PA14_40110		hypothetical protein	4.94	2.30	1.08E-09	6.04E-09
PA14_40520		hypothetical protein	4.54	2.18	4.70E-16	4.46E-15
PA14_40560		hypothetical protein	7.21	2.85	8.85E-69	6.86E-67
PA14_40750		hypothetical protein	4.62	2.21	5.04E-22	6.75E-21
PA14_40780		hypothetical protein	4.27	2.09	8.71E-40	2.76E-38
PA14_41760		hypothetical protein	4.08	2.03	3.03E-19	3.54E-18
PA14_41790		hypothetical protein	5.53	2.47	3.34E-37	9.31E-36
PA14_42410		hypothetical protein	8.04	3.01	7.81E-52	3.77E-50
PA14_44430		hypothetical protein	4.65	2.22	9.04E-43	3.23E-41
PA14_46380		hypothetical protein	4.07	2.03	1.22E-16	1.21E-15
PA14_46390		hypothetical protein	5.20	2.38	5.00E-23	7.12E-22
PA14_47410	*ycgN*	hypothetical protein	4.94	2.31	4.32E-21	5.53E-20
PA14_49850		hypothetical protein	4.11	2.04	6.53E-12	4.56E-11
PA14_51940		hypothetical protein	5.06	2.34	3.20E-21	4.14E-20
PA14_51950		hypothetical protein	4.44	2.15	6.76E-35	1.73E-33
PA14_52910		hypothetical protein	10.54	3.40	2.00E-14	1.69E-13
PA14_54870		hypothetical protein	3.91	1.97	7.75E-39	2.37E-37
PA14_55760		hypothetical protein	3.89	1.96	5.51E-08	2.59E-07
PA14_58010		hypothetical protein	5.29	2.40	7.86E-50	3.56E-48
PA14_60480		hypothetical protein	8.62	3.11	1.49E-72	1.28E-70
PA14_68620		hypothetical protein	4.44	2.15	8.03E-33	1.89E-31
PA14_71190		hypothetical protein	38.36	5.26	5.70E-21	7.25E-20

		**Membrane proteins**				
PA14_02060		hypothetical protein	5.32	2.41	5.75E-04	1.61E-03
PA14_22350	*actP*	acetate permease	5.30	2.41	1.18E-44	4.57E-43
PA14_28400	*oprQ*	outer membrane OprD family porin	12.21	3.61	1.40E-121	2.65E-119
PA14_29660		hypothetical protein	4.22	2.08	3.02E-08	1.46E-07
PA14_32720		transmembrane sensor	5.21	2.38	6.05E-29	1.20E-27
PA14_38950		hypothetical protein	27.61	4.79	9.52E-133	2.16E-130
PA14_39810		transmembrane sensor	5.21	2.38	6.15E-36	1.68E-34
PA14_39830		hypothetical protein	4.34	2.12	2.20E-15	1.99E-14
PA14_40540		hypothetical protein	5.68	2.51	2.39E-33	5.78E-32
PA14_46650		transmembrane sensor	4.63	2.21	1.85E-29	3.73E-28
PA14_64690		transmembrane sensor	4.83	2.27	4.54E-38	1.34E-36
		**Motility & Attachment**				
**Type IV pili**						
PA14_05190	*pilU*	twitching motility protein PilU	4.28	2.10	4.58E-11	2.92E-10
PA14_05340	*pilI*	twitching motility protein PilI	4.18	2.06	1.68E-27	2.98E-26
PA14_05380	*pilK*	methyltransferase PilK	3.92	1.97	8.85E-29	1.71E-27
PA14_58730	*pilA*	type IV pilin structural subunit	3.93	1.97	3.10E-39	9.60E-38
PA14_66630	*pilP*	type 4 fimbrial biogenesis protein PilP	5.09	2.35	1.05E-22	1.45E-21
		**Nucleotide biosynthesis and metabolism**				
PA14_05740	*pydA*	dihydropyrimidine dehydrogenase	31.43	4.97	1.42E-76	1.39E-74
PA14_05770	*dhT*	phenylhydantoinase	6.50	2.70	2.84E-61	1.86E-59
PA14_05790		transporter	6.49	2.70	4.35E-15	3.84E-14
PA14_05810	*amaB*	allantoate amidohydrolase	5.47	2.45	1.21E-31	2.71E-30
PA14_44740	*xdhB*	xanthine dehydrogenase	6.73	2.75	7.95E-51	3.74E-49
PA14_44760		xanthine dehydrogenase accessory factor X	4.49	2.17	1.20E-24	1.86E-23
PA14_53290	*trxB2*	thioredoxin reductase 2	3.87	1.95	5.64E-10	3.27E-09
PA14_61460	*hpt*	hypoxanthine-guanine phosphoribosyltransferase	3.97	1.99	1.12E-15	1.03E-14
PA14_61770	*prs*	ribose-phosphate pyrophosphokinase	3.87	1.95	1.16E-37	3.31E-36
		**Protein secretion/export apparatus**				
PA14_19680		hypothetical protein	9.87	3.30	6.99E-10	4.01E-09
PA14_19690		hypothetical protein	4.97	2.31	8.83E-04	2.40E-03
PA14_40240		ABC transporter ATP-binding protein/permease	4.59	2.20	2.02E-22	2.74E-21
PA14_55790		hypothetical protein	16.50	4.04	4.69E-106	7.26E-104
PA14_55800		hypothetical protein	3.86	1.95	2.25E-21	2.93E-20
PA14_55860		hypothetical protein	7.14	2.84	3.00E-05	1.03E-04
PA14_55880		hypothetical protein	14.68	3.88	1.11E-52	5.53E-51
**Type III secretion system**						
PA14_42350	*pscC*	Type III secretion outer membrane protein PscC precursor	4.09	2.03	4.69E-24	7.14E-23

PA14_42360	*pscB*	type III export apparatus protein	7.15	2.84	2.71E-26	4.56E-25
PA14_42380	*exsD*	hypothetical protein	4.20	2.07	7.78E-14	6.30E-13
PA14_42400	*exsB*	exoenzyme S synthesis protein B	5.53	2.47	1.16E-13	9.17E-13
PA14_42440	*popD*	translocator outer membrane protein PopD precursor	7.20	2.85	7.40E-36	2.01E-34
PA14_42450	*popB*	translocator protein PopB	7.31	2.87	2.12E-39	6.60E-38
PA14_42470	*pcrV*	type III secretion protein PcrV	11.31	3.50	2.19E-37	6.20E-36
PA14_42570	*pscN*	type III secretion system ATPase	21.64	4.44	1.56E-48	6.75E-47
**Type II secretion system**						
PA14_55890	*cpaF2*	type II secretion system protein	10.70	3.42	3.17E-43	1.17E-41
PA14_55900		hypothetical protein	8.51	3.09	2.31E-26	3.92E-25
PA14_55920		type II secretion system protein	7.98	3.00	3.96E-50	1.81E-48
PA14_55930		pilus assembly protein	5.32	2.41	7.30E-17	7.35E-16
PA14_55940		hypothetical protein	42.06	5.39	3.05E-221	2.57E-218
		**Putative enzymes**				
PA14_02435		hypothetical protein	30.64	4.94	2.29E-110	3.85E-108
PA14_11810		aldehyde dehydrogenase	21.05	4.40	3.98E-84	4.69E-82
PA14_30500		acyl-CoA dehydrogenase	4.64	2.21	1.05E-29	2.15E-28
PA14_37530		hydrolase	6.27	2.65	6.98E-51	3.31E-49
PA14_40030		hypothetical protein	100.91	6.66	2.60E-226	2.55E-223
PA14_51050	*pcd*	aldehyde dehydrogenase	5.75	2.52	1.79E-32	4.14E-31
PA14_52900		acyl-CoA dehydrogenase	9.81	3.29	5.24E-36	1.44E-34
PA14_54630		acyl-CoA dehydrogenase	8.86	3.15	9.31E-66	6.69E-64
PA14_54640		enoyl-CoA hydratase	6.88	2.78	3.91E-52	1.90E-50
PA14_54660		enoyl-CoA hydratase/isomerase	7.87	2.98	9.03E-57	5.37E-55
PA14_56840		hypothetical protein	4.80	2.26	1.04E-40	3.46E-39
		**Secreted Factors (toxins, enzymes, alginate)**				
PA14_26020		aminopeptidase	4.24	2.08	3.87E-19	4.49E-18
**Phenazine biosynthesis**						
PA14_09470	*phzB1*	phenazine biosynthesis protein	6.25	2.64	1.21E-18	1.36E-17
PA14_09480	*phzA1*	phenazine biosynthesis protein	6.92	2.79	2.12E-74	1.95E-72
PA14_09490	*phzM*	phenazine-specific methyltransferase	7.15	2.84	1.57E-79	1.68E-77
**Type III secretion system**						
PA14_00560	*exoT*	exoenzyme T	4.26	2.09	1.84E-23	2.70E-22
PA14_36345	*exoY*	adenylate cyclase	7.12	2.83	6.08E-45	2.37E-43
PA14_42430	*exsC*	exoenzyme S synthesis protein C	8.33	3.06	2.02E-23	2.95E-22
PA14_40290	*lasA*	LasA protease	4.94	2.31	1.63E-50	7.48E-49
		**Transcriptional regulators**				
PA14_13150		transcriptional regulator	7.12	2.83	1.13E-71	9.55E-70
PA14_25800		TetR family transcriptional regulator	4.66	2.22	1.12E-41	3.85E-40

PA14_37990		RNA polymerase sigma factor	4.08	2.03	5.18E-28	9.48E-27
PA14_47580		MarR family transcriptional regulator	7.68	2.94	1.42E-33	3.48E-32
PA14_55160	*toxR*	transcriptional regulator ToxR	5.92	2.57	3.58E-32	8.07E-31
PA14_64700		RNA polymerase sigma factor	5.10	2.35	9.66E-51	4.49E-49
PA14_71070		AraC family transcriptional regulator	4.10	2.04	1.25E-30	2.73E-29
**ECF sigma factors**						
PA14_01840		RNA polymerase ECF-subfamily sigma-70 factor	7.08	2.82	1.78E-52	8.83E-51
PA14_32710		ECF subfamily RNA polymerase sigma-70 factor	6.44	2.69	7.10E-66	5.17E-64
PA14_33260	*pvdS*	extracytoplasmic-function sigma-70 factor	9.30	3.22	7.58E-62	5.01E-60
PA14_39800		ECF subfamily RNA polymerase sigma-70 factor	5.85	2.55	9.69E-51	4.49E-49
PA14_46660		RNA polymerase ECF-subfamily sigma-70 factor	8.26	3.05	6.46E-69	5.08E-67
PA14_47400		RNA polymerase ECF-subfamily sigma-70 factor	4.39	2.13	1.40E-39	4.41E-38
**Glycerol metabolism**						
PA14_38930	*glpR*	transcriptional regulator	11.51	3.53	1.60E-100	2.35E-98
**Quorum sensing**						
PA14_39980	*qscR*	transcriptional regulator	9.23	3.21	1.27E-78	1.33E-76
		**Translation, post-translational modification, degradation**				
PA14_13840		peptidyl-prolyl cis-trans isomerase, PpiC-type	4.42	2.14	9.55E-34	2.36E-32
PA14_57580	*rpsI*	30S ribosomal protein S9	4.65	2.22	1.01E-25	1.67E-24
PA14_57590	*rplM*	50S ribosomal protein L13	4.36	2.12	2.40E-45	9.61E-44
PA14_60400	*rpsT*	30S ribosomal protein S20	5.16	2.37	5.28E-28	9.60E-27
		**Transport of small molecules**				
PA14_01860		transmembrane sensor	6.10	2.61	1.15E-19	1.37E-18
PA14_02640	*mcdM*	malonate transporter subunit MadM	4.57	2.19	4.16E-13	3.19E-12
PA14_02690		amino acid permease	5.39	2.43	5.87E-15	5.15E-14
PA14_02700		aminotransferase	26.02	4.70	2.85E-35	7.42E-34
PA14_02720		hypothetical protein	40.01	5.32	2.56E-44	9.87E-43
PA14_16410		MFS transporter	11.10	3.47	1.05E-81	1.17E-79
PA14_18680	*bfd*	hypothetical protein	3.89	1.96	2.89E-07	1.26E-06
PA14_30590		outer membrane receptor protein	4.51	2.17	6.80E-19	7.76E-18
PA14_30600		permease	9.36	3.23	1.69E-46	6.96E-45
PA14_34770		ABC transporter substrate-binding protein	8.23	3.04	3.70E-11	2.37E-10
PA14_34780		ABC transporter ATP-binding protein	5.27	2.40	6.47E-24	9.79E-23
PA14_34790		ABC transporter permease	5.17	2.37	3.46E-14	2.85E-13
PA14_38220		hypothetical protein	4.47	2.16	4.86E-43	1.78E-41
PA14_38320	*yfeH*	bile acid/Na+ symporter family transporter	8.67	3.12	5.06E-77	5.05E-75

PA14_38580		hypothetical protein	6.07	2.60	2.37E-23	3.43E-22
PA14_45240		amino acid permease	4.15	2.05	9.01E-16	8.43E-15
PA14_46910	*ybeJ*	ABC transporter substrate-binding protein	4.26	2.09	5.96E-40	1.90E-38
PA14_51880	*oprD*	basic amino acid, basic peptide and imipenem outer membrane porin OprD precursor	5.27	2.40	2.47E-56	1.43E-54
PA14_53050	*aroP2*	aromatic amino acid transport protein AroP2	6.87	2.78	3.05E-69	2.42E-67
PA14_57990		hypothetical protein	5.29	2.40	3.89E-20	4.75E-19
PA14_64320		hypothetical protein	4.73	2.24	1.64E-22	2.25E-21
PA14_67840		ABC-type amino acid transporter	4.30	2.10	1.78E-20	2.21E-19
PA14_67860		ABC-type amino acid transporter	5.01	2.32	4.73E-23	6.75E-22
PA14_68070		periplasmic binding protein	5.18	2.37	1.80E-07	8.05E-07
PA14_72340	*gltP*	glutamate/aspartate:proton symporter	5.32	2.41	5.06E-23	7.19E-22
PA14_72710		transporter	3.91	1.97	1.08E-23	1.60E-22
PA14_73110		hypothetical protein	9.70	3.28	3.51E-16	3.34E-15
PA14_73120		hypothetical protein	4.55	2.19	3.82E-43	1.41E-41
**Branched-chain amino acid transport**						
PA14_50540	*livM*	leucine/isoleucine/valine transporter permease subunit	4.32	2.11	1.02E-27	1.83E-26
PA14_50550	*livG*	leucine/isoleucine/valine transporter ATP-binding subunit	4.75	2.25	1.09E-29	2.22E-28
PA14_64860	*livF*	ABC transporter ATP-binding protein	13.13	3.71	3.40E-19	3.95E-18
PA14_64870	*braF*	ABC transporter ATP-binding protein	12.53	3.65	6.23E-43	2.25E-41
PA14_64880	*braE*	branched chain amino acid ABC transporter permease	17.08	4.09	2.14E-48	9.21E-47
PA14_64890	*braD*	branched chain amino acid ABC transporter permease	6.71	2.75	1.18E-25	1.95E-24
**C4-dicarboxylate transport**						
PA14_52820	*dctQ*	C4-dicarboxylate transporter	27.85	4.80	3.01E-27	5.31E-26
PA14_52840	*dctP*	C4-dicarboxylate-binding periplasmic protein	15.64	3.97	1.45E-75	1.40E-73
**Iron uptake**						
PA14_02410		TonB-dependent receptor	36.54	5.19	2.79E-93	3.82E-91
PA14_02490	*tonB2*	hypothetical protein	15.54	3.96	3.57E-122	7.02E-120
PA14_02500	*exbB1*	transport protein ExbB	21.18	4.40	2.75E-153	8.11E-151
PA14_02510	*exbD1*	transport protein ExbD	26.20	4.71	1.09E-174	4.57E-172
PA14_09970	*fpvB*	type I ferripyoverdine receptor, FpvB	9.98	3.32	1.80E-87	2.26E-85
PA14_33680	*fpvA*	ferripyoverdine receptor	10.23	3.35	2.37E-106	3.78E-104
PA14_37980		Fe2+-dicitrate sensor, membrane protein	4.60	2.20	2.72E-12	1.96E-11
PA14_39820	*ufrA*	tonB-dependent receptor protein	5.75	2.52	1.34E-30	2.90E-29
PA14_45340		heme exporter protein	5.47	2.45	2.67E-30	5.74E-29

PA14_47380	*hxuC*	heme utilization protein	4.40	2.14	4.31E-39	1.32E-37
PA14_68900	*fbpA*	iron ABC transporter substrate-binding protein	4.38	2.13	7.12E-20	8.63E-19
PA14_72640		TonB-dependent receptor	4.29	2.10	5.84E-40	1.87E-38
**Pyochelin biosynthesis**						
PA14_09270	*pchE*	dihydroaeruginoic acid synthetase	4.03	2.01	2.98E-12	2.14E-11
PA14_09280	*pchF*	pyochelin synthetase	4.00	2.00	5.59E-17	5.68E-16
**Sulfonate transport**						
PA14_02330	*atsC*	ABC transporter ATP-binding protein	29.85	4.90	4.46E-90	5.84E-88
PA14_02340	*atsB*	ABC transporter permease	25.73	4.69	7.96E-151	2.04E-148
PA14_02360	*atsR*	ABC transporter substrate-binding protein	14.31	3.84	1.58E-38	4.76E-37
PA14_12940		taurine ABC transporter ATP-binding protein	5.87	2.55	6.51E-29	1.28E-27
PA14_19510	*ssuC*	ABC transporter permease	4.67	2.22	3.26E-06	1.26E-05
PA14_19580	*ssuB*	aliphatic sulfonates transport ATP-binding subunit	7.35	2.88	4.94E-54	2.67E-52
		**Two-component regulatory systems**				
PA14_06170	*fiuR*	transmembrane sensor	6.41	2.68	2.60E-59	1.60E-57
PA14_38740		two-component sensor	12.02	3.59	6.92E-107	1.13E-104
PA14_47390		transmembrane sensor	6.50	2.70	1.29E-65	9.15E-64
PA14_52250	*pirR*	two-component response regulator	3.91	1.97	6.14E-35	1.59E-33
PA14_57140		two-component response regulator	4.13	2.05	2.30E-20	2.84E-19
**Chemotaxis and motility**						
PA14_02250	*cheA*	two-component sensor	11.17	3.48	2.18E-94	3.14E-92
PA14_05330	*pilH*	twitching motility protein PilH	6.13	2.62	7.45E-55	4.14E-53
**Ethanol catabolism**						
PA14_38900	*exaE*	two-component response regulator	42.47	5.41	4.03E-157	1.32E-154
PA14_38910	*exaD*	sensor kinase	27.54	4.78	1.08E-91	1.45E-89
PA14_38970		two-component sensor	39.20	5.29	9.24E-61	5.91E-59
**C4-dicarboxylate transport**						
PA14_55780		two-component sensor	17.99	4.17	5.12E-48	2.17E-46

[a] If part of an affected pathway or operon, genes differently expressed at least 1.5-fold were also considered.

[b,c,d] Functional categorization, gene ID and gene/product names are taken from the Pseudomonas Genome database[52] and KEGG (Kyoto Encyclopedia of Genes and Genome)[53].

b Transcripts with decreased abundance in B-96 cultures of PA14Δ*hfq* when compared with PA14. A fold change cut-off of -4 and a p-value above 0.05 were applied. The colour coding of different functional classes corresponds to those shown in Fig. 2b.

PA14_id[b]	Gene[c]	Description[d]	Fold Change	log2Fold Change	pval	padj
		**Adaptation, Protection**				
PA14_21760	*capB*	cold acclimation protein B	-4.63	-2.21	4.69E-53	2.40E-51
PA14_27520	*gpo*	glutathione peroxidase	-4.53	-2.18	2.19E-38	6.59E-37
PA14_00710	*osmC*	osmotically inducible protein OsmC	-3.31	-1.72	2.62E-06	1.02E-05
PA14_64480	*osmE*	DNA-binding transcriptional activator OsmE	-2.34	-1.23	2.34E-08	1.14E-07
		**Amino acid biosynthesis and metabolism**				
PA14_02730		dihydrodipicolinate synthetase	-36.75	-5.20	2.23E-75	2.12E-73
PA14_14020		hypothetical protein	-8.35	-3.06	7.39E-42	2.57E-40
PA14_39590	*metE*	5-methyltetrahydropteroyltriglutamate/homocysteine S-methyltransferase	-19.39	-4.28	7.81E-164	2.88E-161
**Histidine degradation**						
PA14_67320	*hutH*	histidine ammonia-lyase	-5.71	-2.51	8.93E-39	2.71E-37
PA14_67350	*hutU*	urocanate hydratase	-4.43	-2.15	8.95E-33	2.10E-31
**Anthranilate biosynthesis**						
PA14_51350	*phnB*	anthranilate synthase component II	-5.38	-2.43	4.48E-45	1.76E-43
PA14_51360	*phnA*	anthranilate synthase component I	-3.92	-1.97	8.87E-27	1.53E-25
		**Biosynthesis of cofactors, prosthetic groups and carriers**				
PA14_01430		short chain dehydrogenase	-6.31	-2.66	4.78E-04	1.35E-03
PA14_06890		hypothetical protein	-5.03	-2.33	9.86E-36	2.62E-34
PA14_39640	*cobN*	cobaltochelatase subunit CobN	-13.18	-3.72	3.12E-06	1.20E-05
PA14_72980		G3E family GTPase	-6.87	-2.78	1.67E-05	5.92E-05
PA14_73010		hypothetical protein	-14.75	-3.88	1.59E-09	8.75E-09
		**Carbon compound catabolism**				
PA14_14300		zinc-binding oxidoreductase	-6.30	-2.66	1.35E-04	4.23E-04
PA14_26130	*morB*	morphinone reductase	-4.74	-2.24	2.46E-42	8.61E-41
PA14_37770		hydrolase	-7.66	-2.94	8.92E-61	5.78E-59
PA14_54000	*prpD*	2-methylcitrate dehydratase	-4.42	-2.14	6.58E-11	4.14E-10
		**Cell wall / LPS / capsule**				
PA14_73040	*amiA*	N-acetylmuramoyl-L-alanine amidase	-11.86	-3.57	9.89E-78	1.02E-75
PA14_61650	*pagL*	Lipid A 3-O-deacylase	-3.10	-1.63	7.98E-22	1.06E-20
PA14_17220	*lpxB*	lipid-A-disaccharide synthase	-2.58	-1.37	9.88E-16	9.19E-15
PA14_20890	*rfaD*	ADP-L-glycero-D-manno-heptose-6-epimerase	-2.31	-1.21	1.32E-09	7.36E-09
PA14_23380	*orfH*	UDP-N-acetyl-D-mannosaminuronate dehydrogenase	-1.68	-0.75	7.95E-05	2.56E-04

PA14_23420		zinc-binding dehydrogenase involved in O antigen biosynthesis	-2.72	-1.44	1.53E-02	3.21E-02
PA14_23430		heparinase	-2.47	-1.31	4.04E-02	7.57E-02
PA14_23450	*orfM*	NAD dependent epimerase/dehydratase	-1.68	-0.75	3.05E-02	5.95E-02
PA14_53500		predicted outer membrane protein and related peptidoglycan-associated (lipo)proteins	-2.14	-1.10	7.80E-11	4.88E-10
PA14_57370	*murD*	UDP-N-acetylmuramoyl-L-alanyl-D-glutamate synthetase	-2.53	-1.34	6.91E-12	4.79E-11
PA14_57390	*murF*	UDP-N-acetylmuramoylalanyl-D-glutamyl-2,6-diaminopimelate--D-alanyl-D-alanine ligase	-2.40	-1.27	1.15E-10	7.10E-10
PA14_57425	*ftsI*	penicillin-binding protein 3	-2.12	-1.08	1.31E-10	8.01E-10
PA14_57450	*mraW*	S-adenosyl-methyltransferase MraW	-2.21	-1.14	3.09E-10	1.84E-09
PA14_62840	*glmM*	phosphoglucosamine mutase	-2.04	-1.03	1.25E-11	8.44E-11
PA14_68170	*rmlB*	dTDP-D-glucose 4,6-dehydratase	-3.38	-1.76	3.53E-10	2.09E-09
PA14_68210	*rmlC*	dTDP-4-dehydrorhamnose 3,5-epimerase	-2.61	-1.39	4.23E-18	4.56E-17
PA14_23460	*orfN*	group 4 glycosyl transferase	-1.78	-0.83	1.59E-02	3.34E-02
		**Central intermediary metabolism**				
PA14_45000	*gcl*	glyoxylate carboligase	-17.13	-4.10	1.34E-122	2.73E-120
PA14_45010	*hyi*	hydroxypyruvate isomerase	-7.79	-2.96	6.89E-24	1.04E-22
		**Chaperones & heat shock proteins**				
PA14_23680	*ibpA*	heat-shock protein IbpA	-4.25	-2.09	4.04E-25	6.44E-24
PA14_42980		ClpA/B-type protease	-9.94	-3.31	4.27E-39	1.32E-37
PA14_62960	*dnaJ*	chaperone protein DnaJ	-3.97	-1.99	1.45E-09	7.98E-09
PA14_62970	*dnaK*	molecular chaperone DnaK	-4.63	-2.21	1.31E-48	5.73E-47
PA14_62990	*grpE*	heat shock protein GrpE	-4.05	-2.02	3.73E-21	4.80E-20
PA14_60190	*clpB*	clpB protein; ATP-binding subunits of Clp protease and DnaK/DnaJ chaperones	-2.82	-1.49	2.85E-22	3.86E-21
PA14_54210	*asrA*	ATP-dependent protease; aminoglycoside-induced stress response ATP-dependent protease AsrA; protein turnover	-2.48	-1.31	4.20E-08	2.00E-07
		**Chemotaxis**				
PA14_46030		chemotaxis transducer	-4.97	-2.31	1.41E-27	2.52E-26
		**DNA replication, recombination, modification and repair**				
PA14_30160	*mutT*	hypothetical protein	-8.07	-3.01	1.00E-29	2.07E-28
		**Energy metabolism**				
PA14_05820		hypothetical protein	-5.85	-2.55	4.04E-11	2.58E-10
PA14_06960		hypothetical protein	-11.83	-3.56	1.86E-93	2.60E-91
PA14_63100		ferredoxin	-8.19	-3.03	2.80E-74	2.54E-72
**Glycerol metabolism**						
PA14_17930	*glpD*	glycerol-3-phosphate dehydrogenase	-4.84	-2.27	1.22E-25	2.00E-24

183

**TCA cycle**						
PA14_30180	*idh*	monomeric isocitrate dehydrogenase	-5.22	-2.38	3.51E-28	6.50E-27
**Lactate oxidation**						
PA14_33860	*lldA*	L-lactate dehydrogenase	-6.22	-2.64	6.10E-29	1.20E-27
PA14_63090	*lldD*	L-lactate dehydrogenase	-10.39	-3.38	9.63E-70	7.88E-68
**Denitrification (*nuo* operon; NADH dehydrogenase)**						
PA14_30020	*nuoA*	NADH dehydrogenase subunit A	-4.48	-2.16	2.27E-35	5.93E-34
PA14_29900	*nuoJ*	NADH dehydrogenase subunit J; NADH-quinone oxidoreductase subunit J	-2.38	-1.25	2.85E-06	1.11E-05
PA14_29920	*nuoI*	NADH dehydrogenase subunit I; NADH-quinone oxidoreductase subunit I	-2.41	-1.27	1.62E-08	7.99E-08
PA14_29930	*nuoH*	NADH dehydrogenase subunit H; NADH-quinone oxidoreductase subunit H	-2.43	-1.28	4.29E-13	3.28E-12
PA14_29970	*nuoF*	NADH dehydrogenase subunit F; NADH-quinone oxidoreductase subunit F	-3.20	-1.68	3.42E-26	5.72E-25
PA14_29940	*nuoG*	NADH dehydrogenase subunit G; NADH-quinone oxidoreductase subunit G	-2.20	-1.14	5.40E-14	4.41E-13
PA14_29980	*nuoE*	NADH dehydrogenase subunit E; NADH-quinone oxidoreductase subunit E	-2.91	-1.54	2.11E-20	2.61E-19
PA14_29990	*nuoD*	bifunctional NADH:ubiquinone oxidoreductase subunit C/D	-3.33	-1.74	8.43E-30	1.76E-28
PA14_30010	*nuoB*	NADH dehydrogenase subunit B; NADH-quinone oxidoreductase subunit B	-2.80	-1.49	8.45E-23	1.18E-21
**Fatty acid and phospholipid metabolism**						
PA14_05840	*gcdH*	glutaryl-CoA dehydrogenase	-10.89	-3.45	1.47E-09	8.08E-09
PA14_11280		hypothetical protein	-13.31	-3.73	3.09E-20	3.78E-19
PA14_16640		lipoprotein	-6.08	-2.60	3.20E-18	3.48E-17
PA14_40800		hypothetical protein	-6.22	-2.64	7.65E-34	1.90E-32
PA14_71890	*psecoA*	coenzyme A transferase	-9.01	-3.17	3.71E-66	2.73E-64
**Hypothetical, unclassified, unknown**						
PA14_06040		hypothetical protein	-4.21	-2.07	3.49E-24	5.35E-23
PA14_10360		hypothetical protein	-5.39	-2.43	4.15E-45	1.64E-43
PA14_11320		hypothetical protein	-15.46	-3.95	3.89E-18	4.20E-17
PA14_12530		hypothetical protein	-5.58	-2.48	9.57E-44	3.57E-42
PA14_15710		hypothetical protein	-4.32	-2.11	4.42E-33	1.05E-31
PA14_16990		hypothetical protein	-12.86	-3.68	3.12E-119	5.74E-117
PA14_18960		hypothetical protein	-5.05	-2.33	6.74E-24	1.02E-22
PA14_28360		hypothetical protein	-6.60	-2.72	3.59E-56	2.05E-54
PA14_31420		hypothetical protein	-5.27	-2.40	1.06E-47	4.47E-46
PA14_32780		hypothetical protein	-6.98	-2.80	9.80E-29	1.89E-27
PA14_37780		hypothetical protein	-11.28	-3.50	6.86E-85	8.42E-83
PA14_38270		hypothetical protein	-4.37	-2.13	2.83E-30	6.03E-29
PA14_39660		hypothetical protein	-8.30	-3.05	5.44E-25	8.61E-24
PA14_43050		hypothetical protein	-5.98	-2.58	1.64E-64	1.12E-62
PA14_44990		hypothetical protein	-6.82	-2.77	3.85E-41	1.30E-39

PA14_47130		hypothetical protein	-5.50	-2.46	2.31E-26	3.92E-25
PA14_53980		hypothetical protein	-5.63	-2.49	1.97E-40	6.44E-39
PA14_54180		hypothetical protein	-26.04	-4.70	3.68E-28	6.78E-27
PA14_56170		hypothetical protein	-7.94	-2.99	9.94E-13	7.36E-12
PA14_58230		hypothetical protein	-6.66	-2.74	3.43E-14	2.83E-13
PA14_58240		hypothetical protein	-4.82	-2.27	5.47E-23	7.71E-22
PA14_60540		hypothetical protein	-3.92	-1.97	4.12E-10	2.43E-09
PA14_60650		hypothetical protein	-5.03	-2.33	3.37E-31	7.45E-30
PA14_63920		hypothetical protein	-48.89	-5.61	1.25E-20	1.56E-19
PA14_63940		hypothetical protein	-61.79	-5.95	1.08E-13	8.59E-13
PA14_68840		hypothetical protein	-5.61	-2.49	5.61E-38	1.65E-36
PA14_73000		hypothetical protein	-19.64	-4.30	3.26E-14	2.69E-13
PA14_73050		GTP cyclohydrolase	-12.25	-3.62	1.01E-28	1.94E-27
		**Membrane proteins**				
PA14_00510		hemagglutinin	-4.69	-2.23	2.12E-40	6.87E-39
PA14_11290		permease	-11.02	-3.46	3.04E-34	7.65E-33
PA14_16630		outer membrane protein, OmpA	-5.22	-2.38	3.61E-29	7.21E-28
PA14_18720	*motY*	OmpA family membrane protein	-4.15	-2.05	1.59E-23	2.35E-22
PA14_26420		TonB-dependent receptor	-4.02	-2.01	3.87E-05	1.31E-04
PA14_54520	*opdH*	porin	-3.90	-1.96	1.13E-14	9.69E-14
PA14_60630		hypothetical protein	-5.29	-2.40	5.29E-35	1.37E-33
PA14_63910		hypothetical protein	-43.91	-5.46	3.51E-24	5.38E-23
PA14_63960		uter membrane protein	-31.62	-4.98	5.17E-23	7.31E-22
PA14_63970		hypothetical protein	-13.76	-3.78	2.20E-13	1.72E-12
		**Nucleotide biosynthesis and metabolism**				
PA14_73070	*pyrQ*	dihydroorotase	-16.06	-4.01	2.60E-17	2.70E-16
		**Protein secretion/export apparatus**				
PA14_10340	*cvaB*	toxin transporter	-4.21	-2.07	3.61E-12	2.58E-11
PA14_10350		secretion protein	-6.81	-2.77	1.97E-18	2.17E-17
**Type VI secretion system**						
PA14_42950		predicted type VI secretion system protein ImpI	-8.75	-3.13	2.32E-10	1.39E-09
PA14_42990		predicted type VI secretion system protein ImpH	-6.28	-2.65	2.70E-41	9.19E-40
PA14_43000		predicted type VI secretion system protein ImpG	-10.94	-3.45	2.67E-66	1.99E-64
PA14_43020		predicted type VI secretion system protein	-6.97	-2.80	2.64E-15	2.37E-14
PA14_43030		predicted type VI secretion system protein ImpC	-7.29	-2.87	5.20E-30	1.10E-28
PA14_43040		predicted type VI secretion system protein ImpB	-5.47	-2.45	5.36E-43	1.95E-41
		**Putative enzymes**				
PA14_09580		hypothetical protein	-6.36	-2.67	7.19E-44	2.73E-42
PA14_09950		oxidoreductase	-8.62	-3.11	8.50E-67	6.50E-65
PA14_17730		hypothetical protein	-8.02	-3.00	5.43E-37	1.51E-35
PA14_37745		carbamoyl transferase	-5.28	-2.40	8.02E-53	4.04E-51
PA14_45020	*glxR*	oxidoreductase	-5.32	-2.41	1.05E-08	5.26E-08
PA14_46620		pyridine nucleotide-disulfide oxidoreductase	-4.55	-2.19	4.71E-44	1.80E-42

PA14_49800	*gsp69*	oxidoreductase	-5.14	-2.36	3.25E-27	5.71E-26
PA14_56160	*moxR*	hypothetical protein	-4.09	-2.03	4.52E-23	6.46E-22
**Secreted Factors (toxins, enzymes, alginate)**						
**Rhamnolipids biosynthesis**						
PA14_19100	*rhlA*	rhamnosyltransferase chain A	-4.97	-2.31	1.03E-53	5.48E-52
PA14_19110	*rhlB*	rhamnosyltransferase chain B	-3.60	-1.85	3.67E-11	2.36E-10
**Transcription, RNA processing and degradation**						
PA14_08760	*rpoB*	DNA-directed RNA polymerase subunit beta	-4.12	-2.04	6.61E-46	2.67E-44
PA14_08780	*rpoC*	DNA-directed RNA polymerase subunit beta'	-9.68	-3.28	3.83E-12	2.73E-11
**Transcriptional regulators**						
PA14_00460	*trpI*	transcriptional regulator TrpI	-4.76	-2.25	8.13E-17	8.17E-16
PA14_01640		LysR family transcriptional regulator	-4.67	-2.22	1.69E-22	2.32E-21
PA14_06970		Cro/CI family transcriptional regulator	-4.84	-2.27	1.25E-29	2.55E-28
PA14_09570		LysR family transcriptional regulator	-4.98	-2.32	7.26E-32	1.63E-30
PA14_23730		LysR family transcriptional regulator	-6.60	-2.72	5.01E-30	1.07E-28
PA14_34880		GntR family transcriptional regulator	-5.54	-2.47	1.89E-49	8.42E-48
PA14_38680		LysR family transcriptional regulator	-6.15	-2.62	8.94E-36	2.39E-34
PA14_42970		transcriptional regulator	-10.50	-3.39	2.19E-12	1.59E-11
PA14_46480		transcriptional regulator	-6.06	-2.60	1.49E-35	3.92E-34
PA14_47520		transcriptional regulator	-6.12	-2.61	1.56E-40	5.16E-39
**Quorum sensing**						
PA14_45960	*lasR*	transcriptional regulator LasR; quorum sensing	-1.31	-0.39	1.48E-02	3.12E-02
PA14_19120	*rhlR*	transcriptional regulator RhlR; quorum sensing	-2.29	-1.19	1.68E-16	1.65E-15
**Denitrification**						
PA14_29620	*norR*	anaerobic nitric oxide reductase transcriptional regulator; activator; denitrification	-2.45	-1.29	8.47E-14	6.83E-13
**Translation, post-translational modification, degradation**						
PA14_17700	*rpmE2*	50S ribosomal protein L31	-9.38	-3.23	2.56E-15	2.31E-14
PA14_17710	*rpmJ*	50S ribosomal protein L36	-7.44	-2.89	1.50E-33	3.65E-32
**Transport of small molecules**						
PA14_11310		ABC transporter ATP-binding protein	-11.66	-3.54	4.74E-18	5.09E-17
PA14_13990		amino acid ABC transporter	-5.31	-2.41	6.03E-53	3.06E-51
PA14_15200		hypothetical protein	-7.59	-2.92	3.02E-34	7.64E-33
PA14_15700		amino acid permease	-4.25	-2.09	2.71E-33	6.52E-32
PA14_18670	*bfrB*	bacterioferritin	-5.62	-2.49	5.45E-56	3.09E-54
PA14_22650		ABC transporter	-6.08	-2.60	1.44E-25	2.36E-24
PA14_24830		glutathione S-transferase	-8.39	-3.07	4.19E-38	1.24E-36
PA14_37760		MFS transporter	-6.43	-2.68	2.13E-54	1.17E-52
PA14_39650	*cirA*	TonB-dependent receptor	-7.73	-2.95	2.91E-19	3.41E-18
PA14_44520	*ydgF*	drug efflux transporter	-25.66	-4.68	2.54E-151	6.80E-149
PA14_44530	*ygdE*	multidrug efflux system protein MdtI	-9.50	-3.25	3.29E-64	2.23E-62

PA14_44820		transthyretin family protein	-8.54	-3.10	3.87E-77	3.94E-75
PA14_44950	*ygfU*	transporter	-10.28	-3.36	2.42E-73	2.16E-71
PA14_49130	*dctA*	C4-dicarboxylate transporter DctA	-3.93	-1.97	6.88E-19	7.84E-18
PA14_63080	*lldP*	L-lactate permease	-6.45	-2.69	1.56E-56	9.09E-55
PA14_67340	*codB*	cytosine/purines uracil thiamine allantoin permease	-5.77	-2.53	1.28E-29	2.60E-28
PA14_73060	*cynT*	hypothetical protein	-13.23	-3.73	1.77E-17	1.85E-16
		**Two-component regulatory systems**				
PA14_21700		two-component sensor	-5.35	-2.42	4.55E-17	4.66E-16
PA14_64410	*ybiI*	hypothetical protein	-9.85	-3.30	1.29E-22	1.78E-21
PA14_73020		DksA/TraR family C4-type zinc finger protein	-21.61	-4.43	3.97E-13	3.04E-12

[a] If part of an affected pathway or operon, genes differently expressed at least 1.5-fold were also considered.

[b,c,d] Functional categorization, gene ID and gene/product names are taken from the Pseudomonas Genome database[52] and KEGG (Kyoto Encyclopedia of Genes and Genome)[53].

52. Winsor, G. L. *et al.* Enhanced annotations and features for comparing thousands of *Pseudomonas* genomes in the *Pseudomonas* genome database. *Nucleic Acids Res.* **44,** D646-53 (2016).

53. Kanehisa, M., Sato, Y., Kawashima, M., Furumichi, M. & Tanabe, M. KEGG as a reference resource for gene and protein annotation. *Nucleic Acids Res.* **44,** D457-62 (2016).

Supplementary Table S3. Strains and plasmids used in this study.

Strain/plasmid	Genotype/relevant features	Source/reference[a]
P. aeruginosa		
PA14	Wild-type	57
PA14*anr-*	Transposon insertion in *anr*; library ID : 26855	57
PA14*rpoN-*	Transposon insertion in *rpoN*; library ID :44482	57
PA14Δ*hfq*	In-frame deletion of the *hfq* gene	58
PA14Δ*crcZ*	In-frame deletion of the *crcZ* gene	This study
PA14Δ*hfq*Δ*crcZ*	Deletion of the *hfq*- and of the *crcZ* genes	This study
E. coli		
DH5α	*F^-, ϕ80dlacZΔM15, Δ(lacZYA-argF)U169, deoR, recA1, endA1,hsdR17(rk^-, mk^+), phoA, supE44, λ^-, thi-1, gyrA96, relA1*	Invitrogen
HB101	*thi-1 hsdS20(rB-, mB-) supE44 recA13 ara-14 leuB6 proA2 lacY1 galK2 xyl-5 mtl-1 rpsL20*	59
**Plasmids**		
pME3087	Suicide vector, ColE1 replicon, IncP-1, Mob; Tc^R	60
pME3087Δ*crcZ*	Suicide vector used for construction of the in-frame deletion of *crcZ*; Tc^R	This study
pME3087Δ*hfq*	Suicide vector used for construction of the in-frame deletion of *hfq*; Tc^R	This study
pRK2013	Helper plasmid, ColE1 replicon, Tra; Km^r	61
pUX-BF13	*mob*$^+$*ori*-R6K; helper plasmid, providing the Tn7 transposition functions *in trans*; Amp^r	62
pBK-mini-Tn7-*rrnB*P1–*gfp*(AGA)	Delivery plasmid for mini-Tn7-*rrnB*P1–*gfp*-AGA-a, Amp^r, Gm^r	24
pMMB*crcZ*	pMMBΔ*rbs* harboring *crcZ* under transcriptional control of P_{tac}	15

[a] Additional references:

57. Liberati, N. T. et al. An ordered, nonredundant library of *Pseudomonas aeruginosa* strain PA14 transposon insertion mutants. *Proc. Natl. Acad. Sci. United States Am*. 103, 2833–2838 (2006).
58. Wurtzel, O. *et al.* The single-nucleotide resolution transcriptome of *Pseudomonas aeruginosa* grown in body temperature. *PLoS Pathog.* **8,** e1002945 (2012).
59. Boyer, H. W. & Roulland-Dussoix, D. A complementation analysis of the restriction and modification of DNA in *Escherichia coli*. *J. Mol. Biol.* **41,** 459–472 (1969).
60. Voisard, C. *et al.* in *Molecular Ecology of Rhizosphere Microorganisms* 67–89 (Wiley-VCH Verlag GmbH, 1994). doi:10.1002/9783527615810.ch6
61. Figurski, D. H. & Helinski, D. R. Replication of an origin-containing derivative of plasmid RK2 dependent on a plasmid function provided in *trans*. *Proc. Natl. Acad. Sci. U. S. A.* **76,** 1648–1652 (1979).
62. Bao, Y., Lies, D. P., Fu, H. & Roberts, G. P. An improved Tn7-based system for the single-copy insertion of cloned genes into chromosomes of Gram-negative bacteria. *Gene* **109,** 167–168 (1991).

Supplementary Table S4. Oligonucleotides used in this study.

Name	Sequence[a]	Restriction site
C3	GCACAACAACAATAACAAGCAACGACGAAG	
C6	ATGC**GGATCC**GAAATGGTGTAAGGCGAAGG	*Bam*HI
D112	GTACAGGGAACACGCAACC	
E2	CTTCTTCCGACTGGCTGCGGG	
E6	**TCTAGA**<u>CGTAATACGACTCACTATAGG</u>CACAACAACAATAA CAAGC	*Xba*I
E112	CCCCGCCCACTCTTCAG	
H71	TTTTTTT**GGATCC**GATCCCGAGGTGCGCGC	*Bam*HI
I26	CCCCACACTACCATCGGCGATGCGTCG	
I71	TTTTTTT**GAATTC**GCGGCAGGTGCG	*Eco*RI
J71	GTGCCGCACTCCTTTAAGG	
K3	GCTGGAGTCGTTACGTGTTG	
K71	CCTTAAAGGAGTGCGGCACTGACGGGAGTCCGCTTTG	
T131	CGAATGGCTTCTTGAGC	
U131	GAGCTTCGTATGGGGAG	
W100	GCATAC**GGTACC**ACCGCGACCTGAAGACCCTGGC	*Kpn*I
X100	GCTCAT**TCTAGA**CAGTTTGAAGCGGCGGCCGATG	*Xba*I
Y100	CAATACATAAGCAGATGCCGTGCCAG	
Z100	CTGGCACGGCATCTGCTTATGTATTGAAACAAAGCACGCACCTACTTGGG	

[a] Restriction sites are highlighted in bold. The T7 promoter sequence is underlined.
